# Genome-wide analysis of the AP2/ERF transcription factor superfamily in Chinese cabbage (*Brassica rapa* ssp. *pekinensis*)

**DOI:** 10.1186/1471-2164-14-573

**Published:** 2013-08-23

**Authors:** Xiaoming Song, Ying Li, Xilin Hou

**Affiliations:** 1 State Key Laboratory of Crop Genetics and Germplasm Enhancement/Key Laboratory of Biology and Germplasm Enhancement of Horticultural Crops in East China, Ministry of Agriculture, Nanjing Agricultural University, Nanjing 210095, China

**Keywords:** Chinese cabbage, AP2/ERF, Stress tolerance, Gene expression, Interaction network, Protein annotation

## Abstract

**Background:**

Chinese cabbage (*Brassica rapa* ssp. *pekinensis*) is a member of one of the most important leaf vegetables grown worldwide, which has experienced thousands of years in cultivation and artificial selection. The entire Chinese cabbage genome sequence, and more than forty thousand proteins have been obtained to date. The genome has undergone triplication events since its divergence from *Arabidopsis thaliana* (13 to 17 Mya), however a high degree of sequence similarity and conserved genome structure remain between the two species. *Arabidopsis* is therefore a viable reference species for comparative genomics studies. Variation in the number of members in gene families due to genome triplication may contribute to the broad range of phenotypic plasticity, and increased tolerance to environmental extremes observed in *Brassica* species. Transcription factors are important regulators involved in plant developmental and physiological processes. The AP2/ERF proteins, one of the most important families of transcriptional regulators, play a crucial role in plant growth, and in response to biotic and abiotic stressors. Our analysis will provide resources for understanding the tolerance mechanisms in *Brassica rapa* ssp. *pekinensis*.

**Results:**

In the present study, 291 putative AP2/ERF transcription factor proteins were identified from the Chinese cabbage genome database, and compared with proteins from 15 additional species. The Chinese cabbage AP2/ERF superfamily was classified into four families, including AP2, ERF, RAV, and Soloist. The ERF family was further divided into DREB and ERF subfamilies. The AP2/ERF superfamily was subsequently divided into 15 groups. The identification, classification, phylogenetic reconstruction, conserved motifs, chromosome distribution, functional annotation, expression patterns, and interaction networks of the AP2/ERF transcription factor superfamily were predicted and analyzed. Distribution mapping results showed AP2/ERF superfamily genes were localized on the 10 Chinese cabbage chromosomes. AP2/ERF transcription factor expression levels exhibited differences among six tissue types based on expressed sequence tags (ESTs). In the AP2/ERF superfamily, 214 orthologous genes were identified between Chinese cabbage and *Arabidopsis*. Orthologous gene interaction networks were constructed, and included seven CBF and four AP2 genes, primarily involved in cold regulatory pathways and ovule development, respectively.

**Conclusions:**

The evolution of the AP2/ERF transcription factor superfamily in Chinese cabbage resulted from genome triplication and tandem duplications. A comprehensive analysis of the physiological functions and biological roles of AP2/ERF superfamily genes in Chinese cabbage is required to fully elucidate AP2/ERF, which provides us with rich resources and opportunities to understand crop stress tolerance mechanisms.

## Background

The AP2/ERF transcription factors superfamily is one of the largest groups of transcription factors in plants, which includes at least one APETALA2 (AP2) domain. According to the number of AP2 domains, and the presence of other DNA binding domains, AP2/ERF can be divided into the ERF, AP2, RAV and Soloist families. The ERF family encodes proteins with a single AP2 domain, while the AP2 gene family codes for transcription factors with two AP2 domains [1-3]. With the exception of a single AP2 domain, however, there is one additional B3 domain in the RAV gene family. The B3 domain is a DNA-binding domain conserved in other plant specific transcription factors [4].

To date, two major schemes have been applied to define the ERF family nomenclature. According to DNA binding domain protein sequences, the ERF family was divided into two major subfamilies, the ERF and DREB subfamilies. ERF and DREB were divided into six groups in *Arabidopsis*[5]. Alternatively, based on AP2/ERF domain amino acid sequences, *Arabidopsis* and rice ERF families were divided into 12 and 15 respective groups [2]. Similarly, 10 groups were identified in the grape and cucumber ERF family [1,4].

Despite high sequence conservation in the AP2/ERF domain, each family exhibits different DNA elements. Generally, the ERF subfamily binds to an AGCCGCC sequence, i.e. the GCC box [6], while the DREB subfamily typically interacts with a CCGAC core sequence [7]. The AP2 family, even with the presence of two AP2 domains, does not bind to the CCGA/CC sequence as in DREB/ERF subfamilies, but binds to the GCAC(A/G)N(A/T)TCCC(A/G)ANG(C/T) element [8,9]. AP2 family genes are regulated by microRNA (miR172), and can be divided into AP2 and ANT groups [10,11]. RAV family binds to the CAACA and CACCTG sequence. Such as pepper CARAV1 can recognize and bind to these motifs, and activate the yeast reporter gene [12].

A variety of AP2/ERF transcription factors have been successfully identified and investigated in some plants, including *Arabidopsis*, rice [2,13], grape [1], poplar (*Populus tricocarpa*) [14], wheat (*Triticum aestivum*) [15], cucumbers [4], barley (*Hordeum vulgare*) [16], and soybean (*Glycine max*) [17]. The AP2/ERF transcription factors regulate diverse biological processes in plant function and development, such as hormones, reproduction, cell proliferation, abiotic and biotic stress responses [18,19].

Commonly, the DREB subfamily is used as viable candidate to enhance crop abiotic stress tolerance. The DREB subfamily exhibits different response patterns under environmental stress, including low-temperature (*AtCBF1*) [20], heat (*ZmDREB2A*, *AtDREB1A*) [21,22], osmotic (*CkDREB*) [23], drought (*OsDREB1*) [24,25], and water-deficit and high-salt stress (*CaDREBLP1*) [26]. The DREB transcription factors activate multiple dehydration/cold-regulated genes by interacting with DRE/CRT elements (A/GCCGAC), which are present in the RD/COR gene promoters [1]. In addition, several DREB subfamily genes are reportedly positive and negative mediators of ABA and sugar responses, primarily during germination and early seedling stages [27].

ERF transcription factors are also involved in signal pathways during environmental stress or pathogen and disease-related stimuli. ERF transcription factors directly regulate pathogenesis-related (PR) gene expression by binding DNA with the GCC-box (GCCGCC), such as PR1 to PR5 [6,28,29]. ERF transcription factors play an important role in plant development, as well as tolerance to biotic and abiotic stress. ERF transcription factor overexpression has been reported in rice [30,31], tomato, and tobacco [32,33], leading to drought and salt tolerant improvements in transgenic plants. Signal molecules, including JA, salicylic acid (SA), ethylene (ET), and abscisic acid (ABA) regulate several important defense-signaling pathways. ERF transcription factors potentially play a role in abiotic and biotic stress in plants, such as drought (*SHN1*, *SHN2* and *SHN3*), salt (*AP37*), freezing (*TaERF1*) [34-36], hypoxic stress (*SNORKEL1*, *SNORKEL2*, *RAP2*.2, *AtERF73* and *HRE1*) [1,37,38], cell dedifferentiation (*WIND1*) [39], metabolite biosynthesis (*LeERF*-1, *Nud*), and trait development (*ORC1*, *ERN* and *EFD)*[40-44]. Most of these ERF transcription factors improve abiotic tolerance in crops without causing undesirable growth phenotypes [18]. However *CRL5*, an AP2 subfamily in rice, promoted crown root initiation in response to ABA [45]. Moreover, *CRL5* affected sepal abscission (*BnAP2*), plant height (*NsAP2*), and leaf shape in *Brassica napus*, water lily, and maize [46-48]. The RAV family was shown to mediate plant defense during abiotic and biotic stress. *CaRAV1* overexpression increased tolerance to high salinity and osmotic stress in *Arabidopsis*, and the *B. napus RAV-1-HY15* gene was induced by cold, NaCl, and PEG treatments [49,50]. These observations emphasize the importance of identifying all AP2/ERF superfamily genes to interpret the mechanisms underlying stress signal transmission, and ultimately manipulate AP2/ERF protein regulation to improve crop stress resistance. ERF-mediated plant defense responses can be better understood by elucidating the signaling pathways involved in defense response regulation.

Chinese cabbage, a member of the genus *Brassica*, is an important leaf vegetable crop grown worldwide. The Chinese cabbage genome (Chiifu-401-42) was recently sequenced and assembled. Data indicated *B. rapa* ssp. *pekinensis* exhibits a close relationship with *A. thaliana*, and experienced a whole genome triplication since its divergence from *Arabidopsis* 13 to 17 Mya [51,52]. The release of the entire Chinese cabbage genome sequence, as well as others, including *Arabidopsis*, potato, and tomato, provided us an opportunity for comparative genome research on AP2/ERF transcription factors. Characterization of AP2/ERF superfamily genes in *B. rapa* ssp. *pekinensis* can serve to clarify the molecular mechanisms responsible for abiotic and biotic stress responses, such as cold, heat, salt, or disease resistance. Subsequently, *Brassica* varieties with increased tolerance to many adverse environments can be developed using transgenic technology. A recent study reported 62 AP2/ERF superfamily genes using expressed sequence tags (ESTs) in Chinese cabbage [53]. In this study, we systematically and comprehensively describe the AP2/ERF transcription factors in *B. rapa* ssp. *pekinensis* through a comparative genome analysis. The objectives of our study were as follows: (i) identify and characterize the AP2/ERF transcription factors in the *B. rapa* ssp. *pekinensis* genome; (ii) analyze AP2/ERF transcription factor phylogenetic relationships and orthologous genes between the *B. rapa* ssp. *pekinensis* and *A. thaliana* genome; and (iii) construct AP2/ERF transcription factor interaction networks, and analyze AP2/ERF transcription factor expression patterns through comparative genomics. ESTs were applied in AP2/ERF transcription factor expression analyses.

## Results

### Identification of the AP2/ERF family transcription factors in Chinese cabbage

Our extensive search for AP2-domain containing proteins identified 291 distinct AP2/ERF putative transcription factors (Additional file 1: Table S1). A total of 248 genes with a single AP2/ERF domain were assigned to the ERF family. Twenty-nine genes were grouped into the AP2 family, twenty of which were identified due to the tandem repeated double AP2/ERF motif. The remaining nine genes (*Br002*, *Br004*, *Br015*, *Br030*, *Br048*, *Br077*, *Br155*, *Br178* and *Br186*) contained only one AP2 domain, however these genes exhibited high similarity with the *Arabidopsis* AP2 family. In fact, the single AP2 domain was similarly reported in *Arabidopsis*, e.g. *At078, At062*, and *At159*. Fourteen genes, with a single AP2/ERF DNA binding domain and a B3 domain, were assigned to the RAV family. The *Br265* gene was not only divergent from the ERF family, but was homologous to *Arabidopsis* Soloist (*At4g13040*). A similar gene was identified in *P. trichocarpa* and named Soloist.

**Figure 1:**
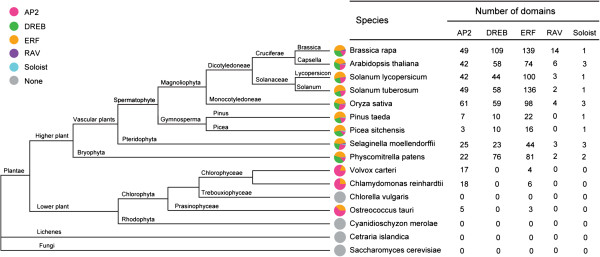
**AP2/ERF transcription factor comparisons among different species.** Different colors represent each family domain in the AP2/ERF superfamily. The colored sections represent the number of transcription factor domains identified in a species. Gray represents the absence of a domain.

We subdivided the Chinese cabbage AP2/ERF genes into 15 groups, based on conserved domain similarities to *Arabidopsis* AP2/ERF transcription factors. Cumulatively, the number of AP2/ERF transcription factors in Chinese cabbage exceeded that in Poplar (202), rice (196), and potato (227). Chinese cabbage AP2/ERF transcription factors were nearly two times that detected in grape (149), tomato (167), and *Arabidopsis* (167). The RAV family number in Chinese cabbage (14) was larger than in other plant groups, including *Arabidopsis* (6), rice (4), tomato (3) and potato (2). The AP2 family domain number was equal in Chinese cabbage (49) and potato, but rice showed more (61), and *Arabidopsis* (42), and tomato (42) fewer, among other species. The Soloist protein, coded by a single-copy gene and characterized by low conservation at the ERF DNA-binding domain, was detected in all higher plant genomes examined. The number of DREB, ERF, and RAV transcription factors in Chinese cabbage exceeded that in each species analyzed. All five AP2/ERF families existed in the higher plants examined, with the exception of *Pinus taeda* and *Picea sitchensis*, where the RAV family was absent. In some lower plants, only the AP2 family and ERF subfamily were identified. AP2/ERF transcription factors were not identified in lichens, fungi, and other lower plants in our analyses (Figure 1).

### Phylogenetic analysis of AP2/ERF transcription factors family

**Figure 2:**
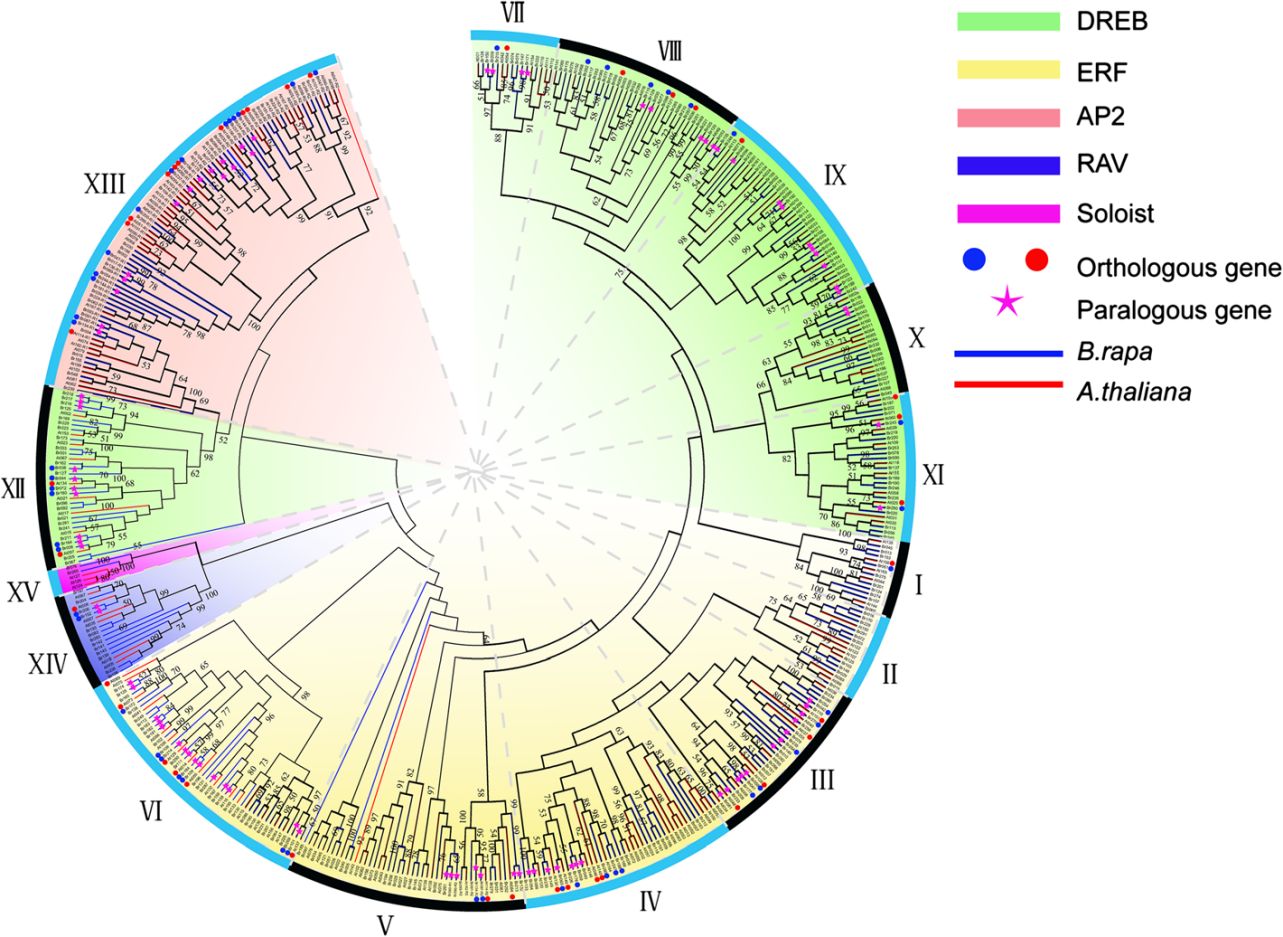
**Phylogenetic tree constructed from the neighbor-joining method using AP2/ERF transcription factor domains in Chinese cabbage and *Arabidopsis*.** The tree was divided into 15 groups, which contained ERF (DREB and ERF subfamily), AP2, RAV and Soloist family. The pentagram represents paralogous genes of Chinese cabbage. Circles represent orthologous genes from Chinese cabbage (blue) and *Arabidopsis* (red). The phylogenetic tree was constructed using MEGA5. The numbers are bootstrap values based on 1000 iterations. Only bootstrap values larger than 50% support are indicated.

The evolutionary relationship between Chinese cabbage and *Arabidopsis* based on AP2/ERF transcription factors was assessed by phylogenetic reconstruction using the conserved AP2/ERF transcription factor domain. The resulting phylogenetic tree (Figure 2) resolved 15 clades, containing the ERF, AP2, RAV and Soloist families, congruent with previous studies [2,5]. Groups I to VI represent the ERF subfamily, and VII to XII the DREB subfamily. Groups XIII, XIV and XV respectively indicate the AP2, RAV and Soloist families. Although the Soloist transcription factor contained a single AP2 domain in Chinese cabbage, it clustered with the RAV family, while the Soloist transcription factor grouped with the AP2 family in grape [1]. We conducted a more in depth phylogenetic analysis of the AP2 family by selecting the AP2 family proteins, which contain two AP2 domains. Chinese cabbage and *Arabidopsis* were divided into two groups, which we named AP2-R1 and AP2-R2. A third group of AP2 transcription factors were formed when a phylogenetic tree was constructed using the AP2 family proteins of all species analyzed (Additional file 2: Figure S1, Figure S2).

The conservative motifs among AP2/ERF proteins in plants were clarified by performing multiple alignment analyses using amino acid sequences of the AP2/ERF domain. For each AP2/ERF family, several genes were selected in one of each species to identify the motifs. Sequence alignment showed the motif length in the RAV family was longest, and contained 50 amino acids, followed by DREB and Soloist (41 amino acids). However, in the AP2 family, 29 amino acids were detected in the motif. The AP2 family contained two groups (AP2-R1, AP2-R2), which might be responsible for the reduced motif length. On the one hand, the divergence between two groups might affect the AP2 family motif length. Generally, the higher the divergence, the shorter the motif. The same explanation might be used for the ERF subfamily (27 amino acids), which contained six groups, and also exhibited a shorter conserved motif. Although six groups were identified in the DREB and ERF subfamilies, the conserved motif was much longer than in the ERF subfamily (Additional file 2: Figure S3). The differences within the AP2/ERF family were further analyzed by examining the DREB, ERF and AP2 conserved motifs using the MEME program. The results showed five of six ERF groups had a 50 amino acid conserved motif, with the exception of the ERFB3 group, which contained 27 amino acids in the conserved motif (Additional file 2: Figure S4). Therefore, if the ERF subfamily motif length was dependent on the ERFB3 group, it might be responsible for the overall shorter ERF subfamily motif. In the DREB subfamily, a shorter conserved motif was observed in DREBA1 compared to the remaining DREB group (50 amino acids), and it showed increased similarity to DREBA3 to DREBA6 groups (Additional file 2: Figure S5). The AP2-R2 group contained 41 amino acids in the conserved motif, and 29 amino acids were identified in the AP2-R1 group conserved motif (Additional file 2: Figure S6).

Sequence alignment of all AP2/ERF families indicated that LG, AA, and YD elements were highly conserved (Additional file 2: Figure S7). The WLG element in DREB, ERF, and RAV was more highly conserved than in AP2 and Soloist (Additional file 2: Figure S3). In the AP2 family, some WLG elements converted into YLG elements, however in the Soloist family, WLG converted into HLG elements. The AYD element was conserved in the AP2/ERF superfamily, with the exception of DREB and Soloist, where it was converted into the AHD element in the DREBA1 and DREBA4 groups. In the Soloist family, the LYD element replaced AYD (Additional file 2: Figure S5, Figure S6).

### Orthologous AP2/ERF genes between Chinese cabbage and *Arabidopsis*

**Figure 3:**
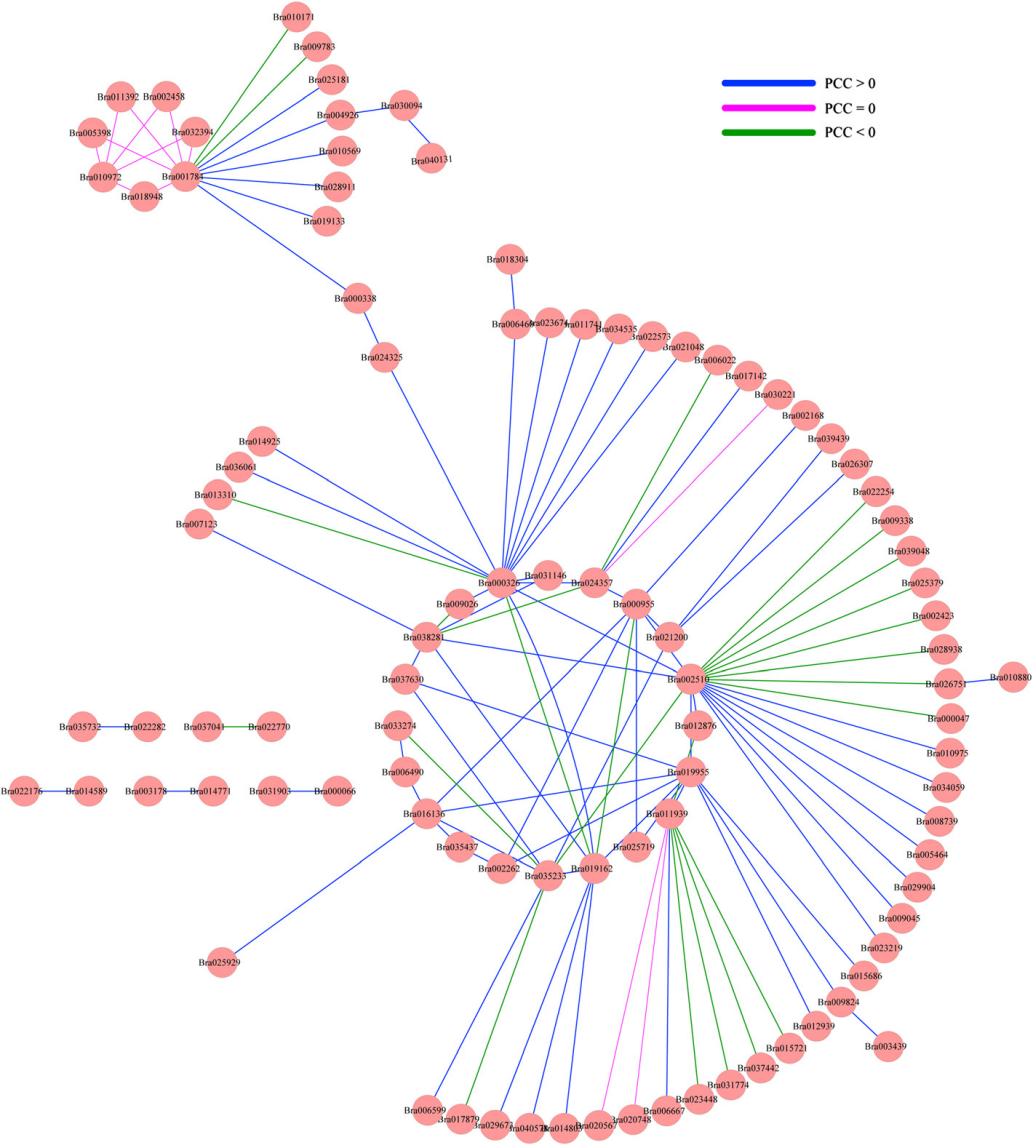
**The interaction network of AP2/ERF in Chinese cabbage according to the orthologs in *Arabidopsis*.** The PCC represents the Pearson Correlation Coefficient.

Comparative genome analysis confirmed Chinese cabbage underwent genome triplication since its divergence from *A. thaliana*. Therefore, many collinear blocks were observed between the Chinese cabbage and *Arabidopsis* genomes. Interestingly, gene number in the Chinese cabbage genome was notably less than three times the *Arabidopsis* gene number. These results indicated gene loss during polyploid speciation in many eukaryotes [52]. Comparative analysis was applied to identify orthologous AP2/ERF transcription factors to assess AP2/ERF gene triplication between Chinese cabbage and *Arabidopsis*; and orthologous genes were shown using the Circos program. Using Blast, we compared AP2/ERF transcription factors, and resolved 214 genes in Chinese cabbage, which exhibited a higher sequence similarity (amino acid identity > 75%) with 128 *Arabidopsis* genes. These results demonstrated that in Chinese cabbage, the AP2/ERF transcription factor duplication accompanied genome triplication (Additional file 2: Figure S8, Additional file 1: Table S2). Results indicated three of the 214 genes anchored in the scaffolds (*Br270-ERF-B1*, *Br288-DREB-A5* and *Br290-DREB-A6*). The accession numbers are respectively *Bra036016*, *Bra040309*, and *Bra040381* in the Chinese cabbage databank. An interaction network was constructed associated with AP2/ERF *Arabidopsis* orthologs using AP2/ERF genes from Chinese cabbage. Pearson correlation coefficient of seventy-seven gene pairs was greater than zero, and twenty-five gene pairs was less than zero (Figure 3). Furthermore, we investigated the Chinese cabbage paralogous AP2/ERF transcription factors. One hundred sixty nine AP2/ERF transcription factors showed high homology (> 80%) to the AP2/ERF proteins (Additional file 2: Figure S9, Additional file 1: Table S3). The orthologous and paralogous genes were indicated in the phylogenetic tree (Figure 2).

Duplication events have been studied in grape, and 17 proteins with high similarity sequences were reported [1]. We identified 15 duplicated genes in the Chinese cabbage genome, with a 95% sequence similarity. Among the 15 duplicated genes, 11 were classified as ERF subfamily, and the remaining were determined DREB subfamily genes (Table 1).

**Table 1:** The gene duplication of AP2/ERF superfamily in Chinese cabbage

**Transcription factor name (*B. rapa *ssp. *pekinensis*)**	**Transcription factor name (*B. rapa *ssp. *pekinensis*)**	**Identity(%)**	**E-value**
Br183-ERF-B3	Br182-ERF-B3	100	2.00E-123
Br183-ERF-B3	Br181-ERF-B3	100	2.00E-123
Br132-ERF-B3	Br213-ERF-B3	100	0
Br261-ERF-B6	Br260-ERF-B6	100	5.00E-53
Br014-DREB-A5	Br154-DREB-A5	97.65	9.00E-60
Br158-ERF-B3	Br159-ERF-B3	100	2.00E-102
Br260-ERF-B6	Br261-ERF-B6	100	9.00E-55
Br181-ERF-B3	Br182-ERF-B3	100	2.00E-123
Br181-ERF-B3	Br183-ERF-B3	100	2.00E-123
Br217-DREB-A4	Br216-DREB-A4	100	8.00E-105
Br217-DREB-A4	Br218-DREB-A4	99.2	1.00E-104
Br111-ERF-B3	Br250-ERF-B3	95.6	1.00E-37
Br159-ERF-B3	Br158-ERF-B3	100	5.00E-102
Br250-ERF-B3	Br111-ERF-B3	95.19	2.00E-46
Br216-DREB-A4	Br217-DREB-A4	100	8.00E-105
Br216-DREB-A4	Br218-DREB-A4	99.2	1.00E-104
Br213-ERF-B3	Br132-ERF-B3	100	0
Br182-ERF-B3	Br181-ERF-B3	100	2.00E-123
Br182-ERF-B3	Br183-ERF-B3	100	2.00E-123
Br218-DREB-A4	Br216-DREB-A4	99.2	8.00E-105
Br218-DREB-A4	Br217-DREB-A4	99.2	8.00E-105

### Chromosome distribution of the AP2/ERF family transcription factors

**Figure 4:**
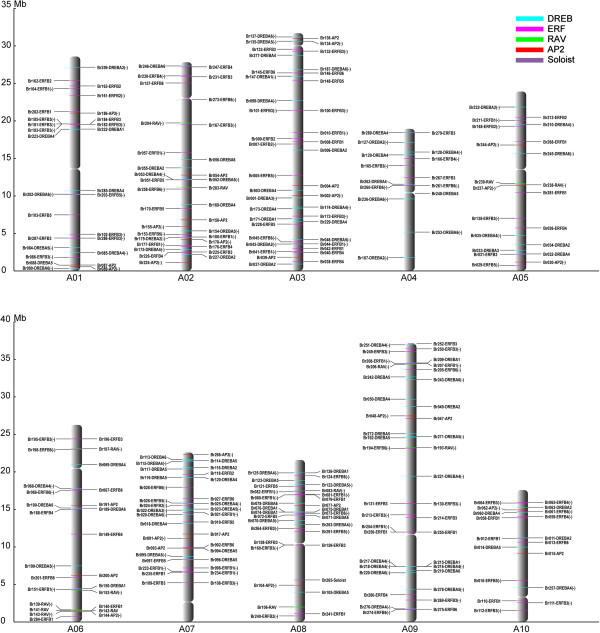
**Distribution of 291 AP2/ERF genes on the 10 Chinese cabbage chromosomes.** The centromeric positions are shown according to Chinese cabbage genome sequencing results. Three genes on the scaffold (*Br270*, *Br288*, and *Br290*) could not be anchored onto a specific chromosome. Scale is in megabases (Mb).

Among all AP2/ERF family transcription factors resolved in the Chinese cabbage genome, 137 genes belong to the ERF subfamily, followed by DREB (107 genes), AP2 (29 genes), RAV (14 genes) and Soloist (1 gene) transcription factors (Table 2). The 288 total AP2/ERF transcription factors were distributed on the 10 Chinese cabbage chromosomes (Figure 4), and three genes could not be assigned to any specific chromosome. Chromosomes 3 and 9 had the highest number of AP2/ERF transcription factors, with 41 and 40 genes, respectively; and the lowest AP2/ERF transcription factor number was found on chromosomes 4 (15 genes) and 10 (18 genes). The high AP2/ERF sequence number on these two chromosomes was primarily due to the increased number of DREB (16 and 18 genes) and ERF (20 and 18 genes) subfamilies. The subfamilies were responsible for 87.8% and 90% of the total AP2/ERF superfamily on chromosomes 3 and 9, respectively. Interestingly, results identified conserved sequences and physical proximity of repetitive transcription factors, which belong to the same group, and were located on the same chromosomal regions, as follows: *Br181* to *Br185* (ERFB3 group), and *Br286* to *Br287* (ERFB3) were located on chromosome 1; *Br233* to *Br235* (ERFB1), and *Br108* to *Br109* (ERFB3) were located on chromosome 7; *Br074* to *Br076* (DREBA1), and *Br158* to *Br160* (ERFB3) were located on chromosome 8; *Br207* to *Br208* (ERFB1), and *Br254* to *Br256* (ERFB1), and *Br274* to *Br275* (ERFB6), and *Br216* to *Br218* (DREBA4) were located on chromosome 9. Similar patterns were also found in the *Arabidopsis*[5], grape, and poplar genomes [1,14], which were suggested to represent paralogous segments resulting from ancestral polyploidization events. The highest RAV transcription factor number was found on chromosome 6 (six genes), followed by chromosomes 2, 5, 8, and 9 (two genes on each chromosome), while RAV transcription factors were not detected on chromosomes 1, 3, 4, 7, and 10.

**Table 2:** Chromosomal distribution of AP2/ERF superfamily in Chinese cabbage

**Chromosome**	**DREB**	**ERF**	**AP2**	**RAV**	**Soloist**	**Total**
A01	9	16	3	0	0	28
A02	10	16	5	2	0	33
A03	16	20	5	0	0	41
A04	9	6	0	0	0	15
A05	7	9	3	2	0	21
A06	6	11	3	6	0	26
A07	15	15	4	0	0	34
A08	12	15	2	2	1	32
A09	18	18	2	2	0	40
A10	5	11	2	0	0	18
Total	107	137	29	14	1	288

### AP2/ERF superfamily transcription factor expression patterns in Chinese cabbage

AP2/ERF transcription factors were integral in plant growth. Therefore, we constructed AP2/ERF transcription factor functional pathways for comprehensive research. The pathway diagrams showed that 65, 54, and 25 AP2/ERF proteins were involved in metabolic and regulatory pathways, and secondary metabolic biosynthesis, respectively (Additional file 2: Figure S10, Figure S11, Figure S12). AP2/ERF protein function in Chinese cabbage was examined by predicting tissue function and expression using ESTs. To date, 168,703 total ESTs, more than 30,000 unique genes, and a corresponding expression profile of Chinese cabbage have been published. These data provided us with rich resources for gene discovery, genome annotation, and studies of gene expression patterns. A total of 174 AP2/ERF transcription factors were obtained by expression profile tags (Additional file 1: Table S4), which contained 96 (55.2%) ERF, 58 (33.3%) DREB, 12 (6.9%) AP2, 7 (4.0%) RAV, and 1 (0.6%) Soloist genes (Table 3, Additional file 2: Figure S13). The expression patterns of these AP2/ERF transcription factors in various tissues, and analysis of the overall similarities and differences among transcriptomes of different tissues or organs were obtained by performing coordinated gene expression analyses of Chinese cabbage ESTs derived from different tissue types. The AP2/ERF transcription factors were detected in six tissues, including buds, flowers, leaves, roots, seeds, and siliques. AP2/ERF transcription factor transcripts were most abundant in roots 42,027 (31.95%), followed by seeds 30,120 (22.90%), leaves 26,384 (20.06%), and flowers 17,288 (13.14%). Few AP2/ERF superfamily genes were detected in siliques 8,051 (6.12%) and buds 7,677 (5.84%) (Additional file 2: Figure S14).

**Table 3:** The expressed number of AP2/ERF genes in six tissues

**Type**	**Bud**	**Flower**	**Leaf**	**Root**	**Seed**	**Silique**	**Type-num**	**Percentage (%)**
AP2	6	8	4	8	3	2	12	6.9
DREB	12	17	35	41	27	13	58	33.3
ERF	31	38	70	75	64	22	96	55.2
RAV	0	5	6	7	7	7	7	4.0
Soloist	1	0	0	0	0	0	1	0.6
Total	50	68	115	131	101	44	174	100

**Figure 5:**
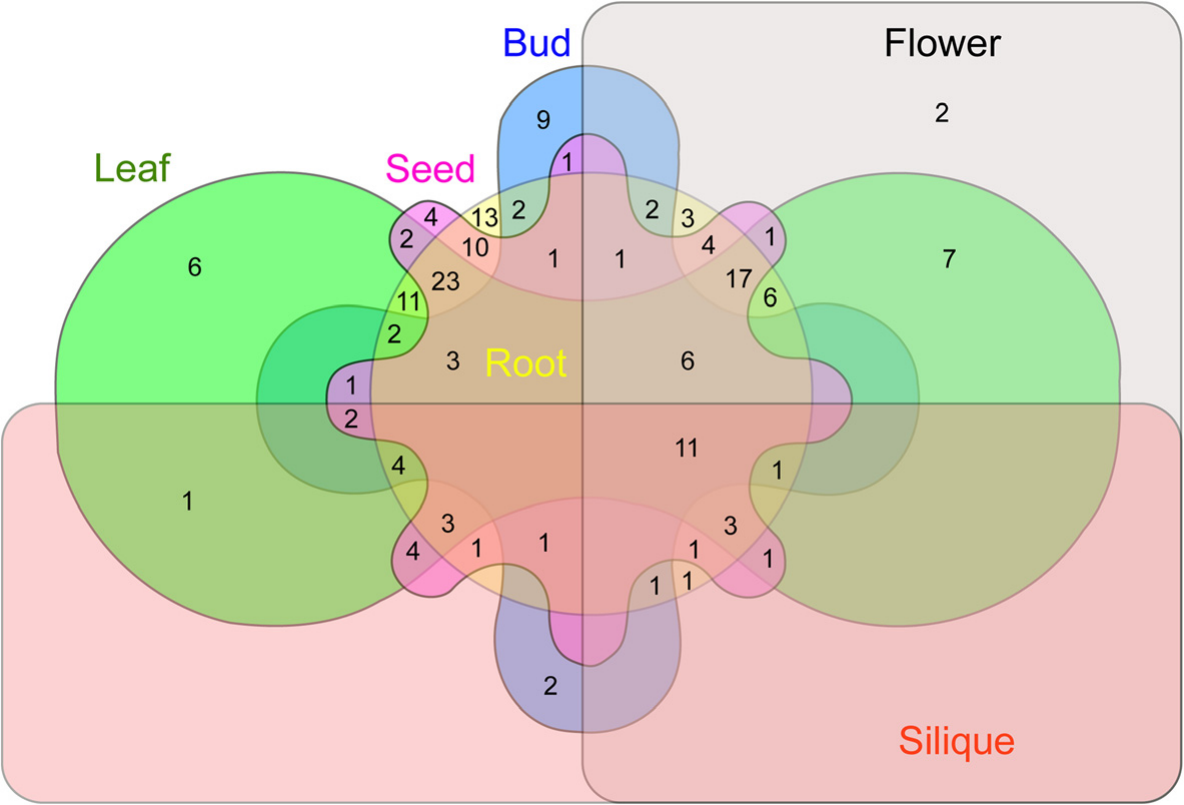
Venn diagram depicting the distribution of shared expression AP2/ERF genes among six Chinese cabbage tissues, i.e. leaves, roots, buds, flowers, siliques, and seeds.

**Figure 6:**
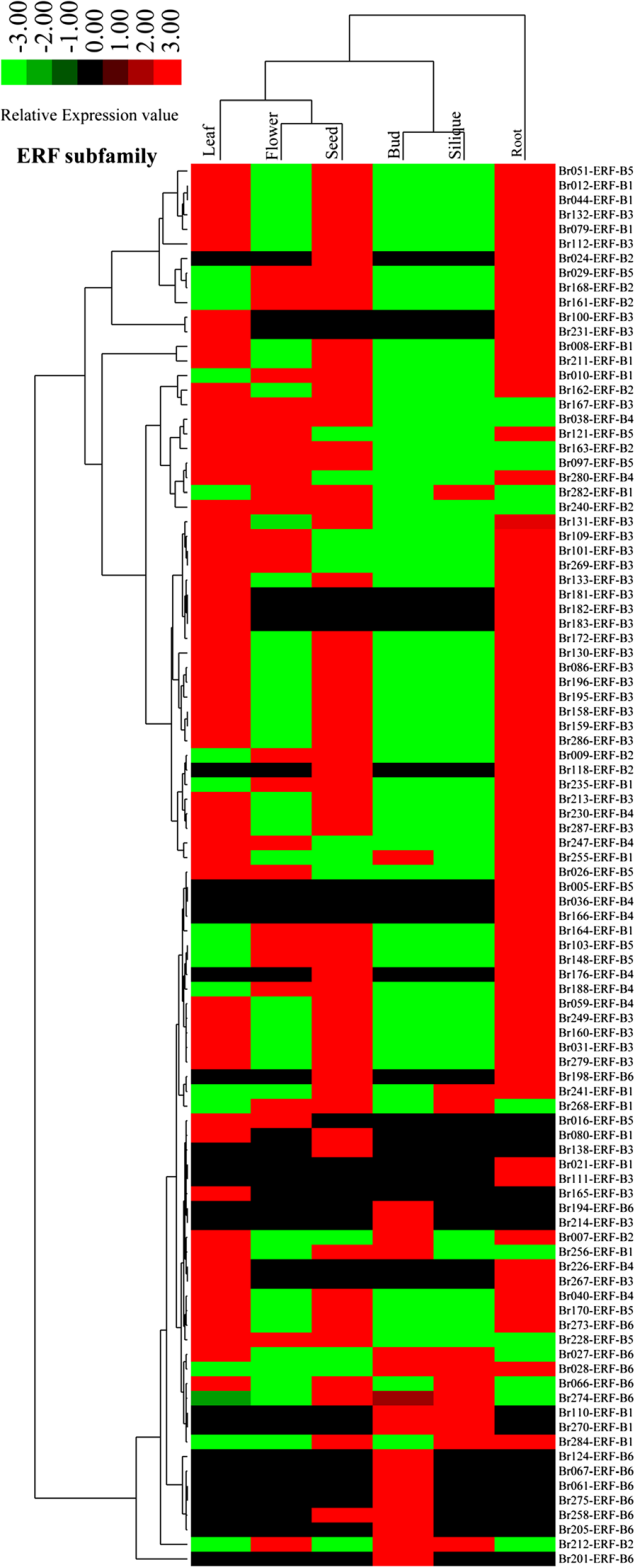
**Expression profile cluster analysis of the Chinese cabbage ERF subfamily proteins.** The expression values of each ERF subfamily gene identified in the study was measured by EST tags from six tissues, i.e. leaves, roots, buds, flowers, siliques, and seeds.

The 174 AP2/ERF transcription factors were detected in some tissues but not others (Figure 5). The highest number of transcription factors was found in coexisting in leaves, roots and seeds (23 genes, 13.22%), followed by genes coexisting in flowers, leaves, roots and seeds (17 genes, 9.77%), and in roots (13 genes, 7.47%) (Additional file 1: Table S5). Furthermore, 11 (6.32%) genes were identified in all six tissues, which contained eight ERF genes, and three DREB genes. In which, the *Br163-ERF-B2* and *Br240-ERF-B2* showed the higher expression in flower than other genes. Gene number in buds, flowers, leaves, roots and seeds was respectively 9, 2, 6, 13 and 4, however gene expression was not detected in siliques. AP2/ERF gene expression in the six tissues varied; expression peaks were detected for the *Br112-ERF-B3* gene in the roots. The DREB subfamily exhibited the highest expression in siliques for the *Br001-DREB-A3* gene, while it was not detected in leaves and seeds. The *Br008-ERF-B1* showed the highest expression level in leaves and seeds compared to the other genes. Surprisingly, the *Br201-ERF-B6* gene showed the highest expression levels in buds compared to other genes and tissues. Especially, it was not expressed in other five tissues. Results showed high expression in seeds for the RAV family, which demonstrated RAV transcription factors were primarily related to fruit development. Expression levels of AP2 transcription factors were lower than most other AP2/ERF genes, and mainly expressed in flowers, buds, seeds, and roots. Detailed expression values and clusters of each AP2/ERF transcription factor were analyzed using cluster analysis based on EST tags from each tissue type (Figure 6, Additional file 2: Figures S15-S18).

### AP2/ERF protein annotations and interactions among specific proteins

**Figure 7:**
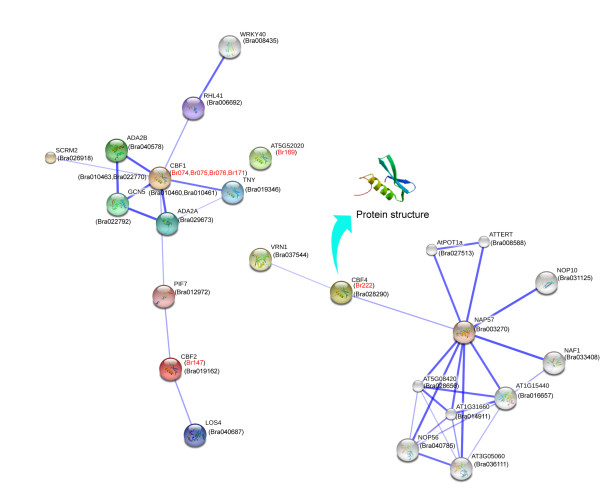
**Interaction network of six CBF genes identified in Chinese cabbage and related genes in *Arabidopsis*.**

**Figure 8:**
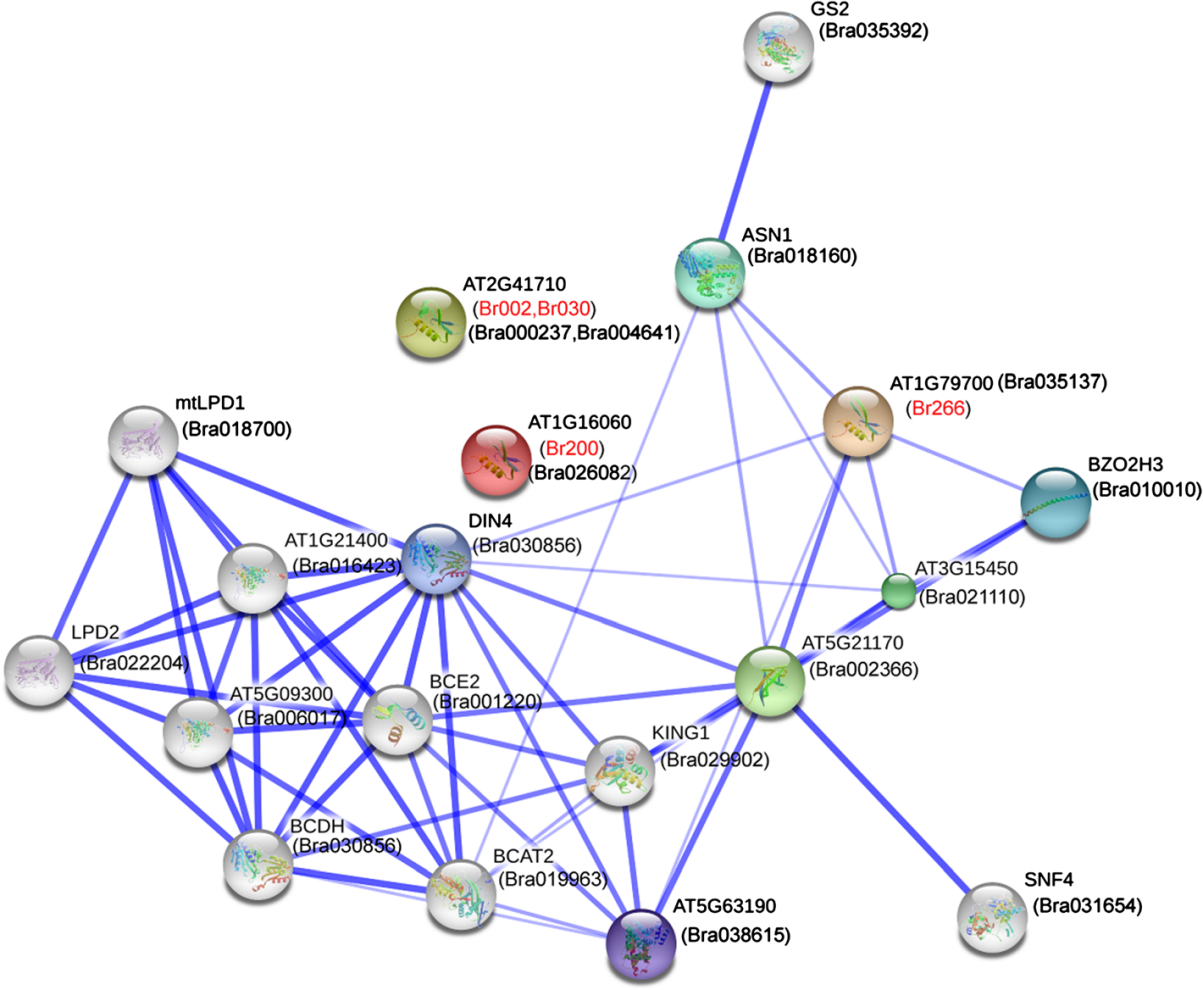
**Interaction network of four AP2 genes associated with ovule development identified in Chinese cabbage and related genes in *Arabidopsis*.**

The predicted Chinese cabbage AP2/ERF superfamily proteins were annotated based on alignment to TrEMBL, Iprscan, SwissProt, GO and KEGG databases using BLASTP at an E value of 1 × 10^−5^. Each AP2/ERF protein annotation from the five protein databases was integrated, and results provided in a supplemental file (Additional file 1: Table S6). Most AP2/ERF proteins belong to the ERF and DREB subfamily. It is interesting we identified seven CBF-like proteins according to the Arabidopsis functional information, which might be related to freezing tolerance in Chinese cabbage. Six of the seven genes were identified as the DREBA1 group, and one protein was classified with the DREBA4 group. Subsequently, Chinese cabbage and *Arabidopsis* protein interactions, including functional and physical interactions were examined used STRING software and the corresponding database to retrieve the among protein interactions. Five proteins, which exhibited increased sequence similarity with *CBF1* (*Br074*, *Br075*, *Br076* and *Br171*) and *CBF2* (*Br147*) were involved in one interaction network. The *Br222* gene, which showed high homology with *CBF4* was involved in another network with the VRN1 protein (Figure 7). The former network largely participated in cold regulatory pathways, as most factors affected cold stress, including *PIF7*, *LOS4*, *CBF1*, *CBF2*, and *ADA2A*[54-57]. Former research suggests the *VRN1* and *CBF4* network might be involved in vernalization, flowering time, or drought adaptation [58,59]. We also found four proteins (*Br002*, *Br030*, *Br200* and *Br266*) in the AP2 family associated with ovule development; and *Br266* interacted with *ASN1*, *BZO2H3* and *DIN4* (Figure 8).

## Discussion

Chinese cabbage is a member of the *Brassica* genus, and *B. rapa* crops are widely used as vegetables, oilseed, condiments, and fodder. Given the important economic value of the species, and its close relationship to *A. thaliana*, the Chinese cabbage genome has been recently sequenced and assembled. *Arabidopsis*, a dicotyledonous species, was the first taxon with its whole genome sequenced and released. Subsequently, the entire genomes of more and more taxa were sequenced, such as the dicot species Chinese cabbage, potato and tomato, and the monocot species rice, among many other plant taxa. These data provided us with rich resources for comparative genomic analyses. Furthermore, with the rapid development in bioinformatics analyses, the information stored in various plant genomes could be explored to elucidate the mechanisms regulating plant growth and development. Plant genome analysis could also facilitate genome and gene evolution studies.

Recent research in structural and functional genomics in higher plant model species e.g. *Arabidopsis* and rice [2] have shown thousands of transcription factors are involved in stress response and plant development. The AP2/ERF genes are viable candidates to improve abiotic and biotic stress in plants, including cold, heat, drought, salt, fungal, and bacterial pathogens. For example, some ERF proteins exhibit resistance to a wide range of pathogens. Furthermore, the AP2/ERF transcription regulators are involved in plant metabolite biosynthesis and trait development, e.g. flowers and roots [18]. However, the AP2/ERF gene characters in Chinese cabbage remain unknown. Therefore, it is essential to identify and annotate the new AP2/ERF genes in Chinese cabbage. In the present study, we identified all AP2/ERF transcription factors in the whole Chinese cabbage genome, and characterized the transcription factors expression patterns in six different tissues.

A comparison of species homologs, including sequenced genomes and expression profiles, might aid in understanding the role of these transcription factors in Chinese cabbage. We assume transcription regulators within the same taxonomic group exhibit recent common evolutionary origins, and specific conserved motifs related to molecular functions. We use this assumption as an effective and practical means to predict unknown protein functions, derived from structural relationships in *Arabidopsis*[2]. Due to the close relationship between Chinese cabbage and *Arabidopsis*, the highly homologous genes were identified and used to predict functions in Chinese cabbage. Finally, we identified five AP2/ERF genes, which showed high similarity (> 90%) with corresponding genes in *Arabidopsis* (Additional file 1: Table S2). Following the homologous gene annotations in *Arabidopsis*, we determined the five AP2/ERF gene functional roles in Chinese cabbage. For example, the *Br270-ERF-B1* transcription factor, which exhibited high sequence similarity with the *At3g15210* gene, likely act as a negative regulator of JA-responsive defense gene expression, and antagonizes JA inhibition of root elongation [60]. The annotation of *Br214-ERF-B3* (homology with *At1g04370* and *At5g43410* genes) showed it is a disease resistance gene [61], and *Br012-ERF-B1* (homology with *At5g13910* genes) was a positive regulator of gibberellic acid-induced germination [62], while *Br008-ERF-B1* (homology with *At5g44210* gene) is a negative regulator of transcription [63]. The *Br047-AP2* (homology with *At3g54320* gene) is involved in storage compound biosynthesis control, and the mutants had a wrinkled seed phenotype [64].

In the present study, we analyzed the AP2/ERF transcription factor superfamily in Chinese cabbage and 15 other species, representing red and green algae, non-vascular and vascular plants, lichens, and fungi. In higher plants, most AP2/ERF genes have been assigned to the AP2 and ERF families. The ERF subfamily gene number exceeded DREB and AP2 genes. However in lower plants, the number of AP2 family genes was more than other transcription factors (i.e. ERF, DREB, RAV, and Soloist). For the 16 species we examined, AP2/ERF transcription factors were not detected in fungi, lichens, and two algal species (i.e. *Chorella vulgaris* and *Cyanidioschyzon merolae*).

We conducted a comprehensive search for AP2/ERF transcription factors throughout the Chinese cabbage genome, and identified 291 genes. A previous study reported 62 AP2/ERF transcription factors using ESTs [53]. Compared to the species considered in this study, the Chinese cabbage genome supports large ERF and DREB subfamilies. The greatest number of AP2 transcription factors has been identified in rice (61) relative to other species, however the number was similar among Chinese cabbage (49), *Arabidopsis* (42), tomato (42) and potato (49). RAV family genes have been determined highly conserved among dicot species, which generally contain six members [1]. However, we identified 14 RAV family genes in Chinese cabbage, and three and two genes assigned to the RAV family in tomato and potato, respectively. Comparative and phylogenetic analyses of AP2/ERF transcription regulators in Chinese cabbage and other species served as a first step in comprehensive functional characterization of AP2/ERF transcription factors by reverse genetic approaches and molecular genetics research.

## Conclusions

In the present study we identified 291 AP2/ERF transcription factors in the Chinese cabbage genome. Isolation and identification of these functional and transcription factor genes are likely to assist in clarifying the molecular genetics basis for Chinese cabbage genetic improvement, and also provide the functional gene resources for transgenic research. These data also constructed the gene network that portrays the control of Chinese cabbage development. To date, few genes representing this transcription factor superfamily have been characterized in detail from Chinese cabbage. Therefore, this is the first comprehensive and systematic research in Chinese cabbage AP2/ERF transcription factors. In silico analyses may assist in elucidating AP2/ERF family gene function in protein interactions, signaling pathway regulations, and defense responses under different stress conditions. Furthermore, it also might provide new opportunities to discover Chinese cabbage tolerance mechanisms under stress conditions. The AP2/ERF superfamily bioinformatics analysis results provided basic resources to examine the molecular regulation of Chinese cabbage development and stress resistance. In addition, the comparative study between Chinese cabbage and other species generated valuable information to study AP2/ERF transcription factor function for economic, agronomic, and ecological benefit in Chinese cabbage.

## Methods

### AP2/ERF superfamily transcription factor identification

Whole genome proteins of several species were downloaded, including Chinese cabbage (http://brassicadb.org/brad/index.php), *Arabidopsis* (http://www.arabidopsis.org/), rice (http://rice.genomics.org.cn), tomato (http://solgenomics.net/organism/Solanum_lycopersicum/genome), and potato (http://potatogenomics.plantbiology.msu.edu/index.html). The following strategy was used to isolate each AP2/ERF superfamily gene from the whole genome of each species. First, the domain types of all proteins were identified using the Pfam program (http://pfam.sanger.ac.uk/) [65]. The AP2/ERF proteins were subsequently selected, and the e-value was set at 1x10^-4^ using the perl program. Third, as a final quality check, we confirmed the presence of the AP2 domain in every AP2/ERF superfamily transcription factor using the SMART database (http://smart.embl-heidelberg.de/) [66]. The sequences of all AP2/ERF superfamily members in the genome of other species assessed were downloaded from the plant TFDB database (http://planttfdb.cbi.edu.cn/) [67]. The *Arabidopsis* AP2 domain for each group defined by Nakano [2] was used as a query to search the AP2/ERF gene domains in Chinese cabbage and other species in the genome database using BLAST. We subsequently obtained the AP2/ERF genes for each species. Each subfamily motif was identified using the MEME program (http://meme.sdsc.edu/meme/intro.html) [68]. The physical distribution of AP2/ERF genes on chromosomes was drawn by perl scripts based on gene position in the genome.

### Phylogenetic tree construction

Phylogenetic and molecular evolutionary analyses were conducted using MEGA5 (http://www.megasoftware.net/) [69]. The retrieved conserved domains of AP2/ERF proteins were used to construct phylogenetic trees. The neighbor-joining method was applied to construct different AP2/ERF transcription factor domain trees, using the pair-wise deletion option. Tree reliability was assessed using 1000 bootstrap replicates. The numbers indicated for each clade represent bootstrap support values given as percentages.

### AP2/ERF superfamily transcription factor expression patterns in Chinese cabbage

Chinese cabbage unigenes and tissue expression level data were downloaded from NCBI (http://ncbi.nih.gov/repository/UniGene/Brassica_rapa/). The AP2/ERF superfamily CDS (coding domain sequence) was extracted from Chinese cabbage, which was used to search against the Chinese cabbage EST database using the BLAST tool. The eligible hits (E-value <1e-5, Identity >90%) were selected for each Chinese cabbage AP2/ERF superfamily transcription factor. Finally, expression levels were calculated in each tissue type (i.e. leaves, roots, buds, flowers, seeds, and siliques) for the AP2/ERF proteins in Chinese cabbage based on the number of eligible hits. The AP2/ERF protein expression cluster from each tissue was analyzed via the Cluster program (http://bonsai.hgc.jp/~mdehoon/software/cluster/software.htm), and results were shown using Tree View software (http://jtreeview.sourceforge.net/).

### Identification of orthologous AP2/ERF genes in Chinese cabbage and *Arabidopsis*

AP2/ERF genes in Chinese cabbage and *Arabidopsis* were compared to identify orthologous genes. Chinese cabbage AP2/ERF genes were used as a query to search against a database built using *Arabidopsis* AP2/ERF genes. The e-value was set at 1e-10, and the identity exceeded 75%. The orthologous AP2/ERF genes between Chinese cabbage and *Arabidopsis* were identified using the Circos program [70]. The AP2/ERF genes in Chinese cabbage were searched for duplication events (e-value < 1e-10, identity > 80%).

### AP2/ERF protein annotations and interaction networks

AP2/ERF protein annotations in *B. rapa* ssp. *pekinensis* were predicted using the protein database TrEMBL (http://www.ebi.ac.uk/uniprot/TrEMBLstats/), Iprscan (http://www.ebi.ac.uk/Tools/pfa/iprscan/), UniProtKB (http://www.ebi.ac.uk/uniprot/), GO (http://www.geneontology.org/), and KEGG (http://www.genome.jp/kegg/); the BLASTP E-value was set as 1 × 10^−5^. AP2/ERF protein annotations in five protein databases were integrated using the perl script. Specific protein interactions were constructed applying STRING software (Search Tool for the Retrieval of Interacting Genes/Proteins, http://string-db.org/) [71]. The functional pathways involving AP2/ERF superfamily were constructed by iPath2.0 software (http://pathways.embl.de/) [72]. The interaction network associated with AP2/ERF *Arabidopsis* orthologs of AP2/ERF genes in Chinese cabbage was constructed using the *Arabidopsis* interaction viewer and cytoscape software [73].

## Competing interests

The authors declare that they have no competing interests.

## Authors’ contributions

The study was conceived by XS and XH. XS collected the public dataset of Chinese cabbage and other species researched. XS contributed to data analysis, bioinformatics analysis, and manuscript preparation. YL and XH participated in planning of analyses and revising the manuscript. All authors had read and approved the final version of the manuscript.

## Supplementary Material

Additional file 1: Table S1 Complete list of ERF/AP2 transcription factors identified in the Chinese cabbage genome. **Table S2.** The orthologue genes of AP2/ERF superfamily between Chinese cabbage and Arabidopsis**. Table S3.** The paralogue genes of AP2/ERF superfamily in Chinese cabbage. **Table S4.** Tissue specific expression of the Chinese cabbage AP2/ERF superfamily genes. The expression profile suggested by analysis of EST counts. **Table S5.** Summary of the Chinese cabbage AP2/ERF superfamily genes expression among the different tissues. **Table S6.** The annotations of all the AP2/ERF proteins of Chinese cabbage in four protein database. Click here for file

Additional file 2: Figure S1 Phylogenetic tree constructed from the neighbor-joining method using AP2 family transcription factor domains in Chinese cabbage and *Arabidopsis*. The numbers are bootstrap values based on 1000 iterations. Only bootstrap values larger than 50 are indicated. **Figure S2.** Phylogenetic tree constructed from the neighbor-joining method using AP2 family transcription factor domains in all 16 species analyzed. **Figure S3.** AP2/ERF protein motifs from each of the species examined. **Figure S4.** The ERF subfamily protein motifs derived from each species examined. **Figure S5.** The DREB subfamily protein motifs derived from each species. **Figure S6.** The RAV, AP2 and Soloist family protein motifs derived from each species examined. **Figure S7.** The AP2/ERF superfamily protein motifs derived from each species examined. **Figure S8.** Comparative analysis of synteny and expansion of AP2/ERF genes. Ten Chinese cabbage and five *Arabidopsis* chromosome maps were based on the orthologue pair positions, and demonstrate highly conserved synteny. **Figure S9.** Comparative analysis of synteny and expansion of AP2/ERF genes. Ten Chinese cabbage chromosome maps were based on the paralogue pair positions; and demonstrate highly conserved synteny. **Figure S10.** The secondary metabolic biosynthesis pathways of the AP2/ERF proteins. **Figure S11.** The regulatory pathways of the AP2/ERF proteins. **Figure S12.** The metabolic pathways of the AP2/ERF proteins. **Figure S13.** AP2/ERF transcription factors classification in Chinese cabbage. The size of each section is proportional to the relative abundance of the AP2/ERF genes assigned to the specific family. **Figure S14.** Distribution of AP2/ERF transcription factors in various Chinese cabbage tissues. **Figure S15.** Expression profile cluster analyses from Chinese cabbage DREB subfamily genes. **Figure S16.** Expression profile cluster analyses from Chinese cabbage RAV family genes. **Figure S17.** Expression profile cluster analyses from Chinese cabbage AP2 family genes. **Figure S18.** Chinese cabbage AP2/ERF superfamily gene expression in six tissue types. Click here for file
