# Supplementary figures and images for: Genome-wide analysis of the AP2/ERF transcription factor superfamily in Chinese cabbage (Brassica rapa ssp. pekinensis)
Source: BMC Genomics. 2013 Aug 23;14:573. doi: 10.1186/1471-2164-14-573 (PMC3765354; doi:10.1186/1471-2164-14-573)

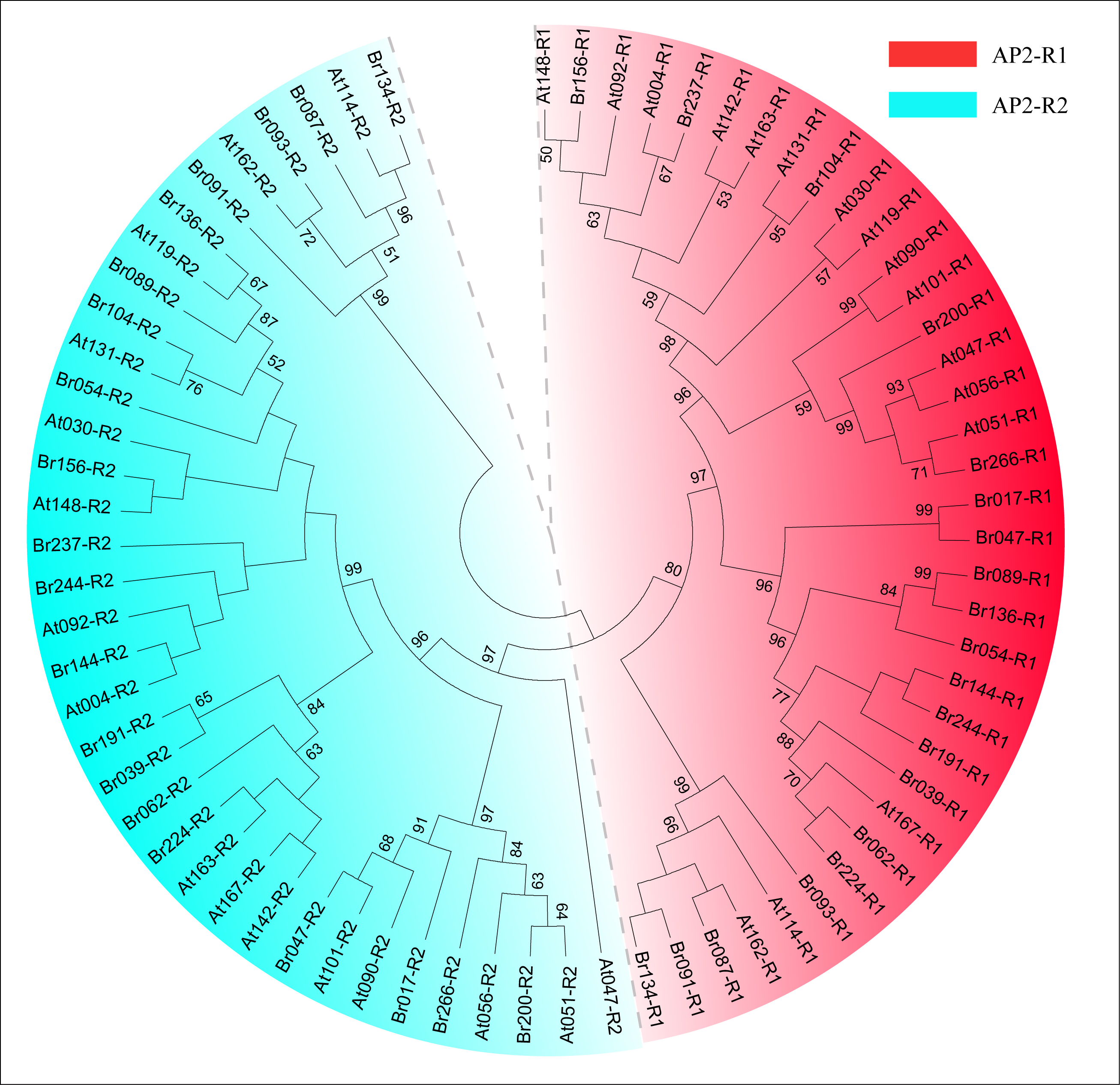

Supplement: Additional file 2: Figure S1 — Phylogenetic tree constructed from the neighbor-joining method using AP2 family transcription factor domains in Chinese cabbage and Arabidopsis. The numbers are bootstrap values based on 1000 iterations. Only bootstrap values larger than 50 are indicated. Figure S2. Phylogenetic tree constructed from the neighbor-joining method using AP2 family transcription factor domains in all 16 species analyzed. Figure S3. AP2/ERF protein motifs from each of the species examined. Figure S4. The ERF subfamily protein motifs derived from each species examined. Figure S5. The DREB subfamily protein motifs derived from each species. Figure S6. The RAV, AP2 and Soloist family protein motifs derived from each species examined. Figure S7. The AP2/ERF superfamily protein motifs derived from each species examined. Figure S8. Comparative analysis of synteny and expansion of AP2/ERF genes. Ten Chinese cabbage and five Arabidopsis chromosome maps were based on the orthologue pair positions, and demonstrate highly conserved synteny. Figure S9. Comparative analysis of synteny and expansion of AP2/ERF genes. Ten Chinese cabbage chromosome maps were based on the paralogue pair positions; and demonstrate highly conserved synteny. Figure S10. The secondary metabolic biosynthesis pathways of the AP2/ERF proteins. Figure S11. The regulatory pathways of the AP2/ERF proteins. Figure S12. The metabolic pathways of the AP2/ERF proteins. Figure S13. AP2/ERF transcription factors classification in Chinese cabbage. The size of each section is proportional to the relative abundance of the AP2/ERF genes assigned to the specific family. Figure S14. Distribution of AP2/ERF transcription factors in various Chinese cabbage tissues. Figure S15. Expression profile cluster analyses from Chinese cabbage DREB subfamily genes. Figure S16. Expression profile cluster analyses from Chinese cabbage RAV family genes. Figure S17. Expression profile cluster analyses from Chinese cabbage AP2 family genes. Figure S18. Chines [file 1471-2164-14-573-S2.zip › Figure S1.png]

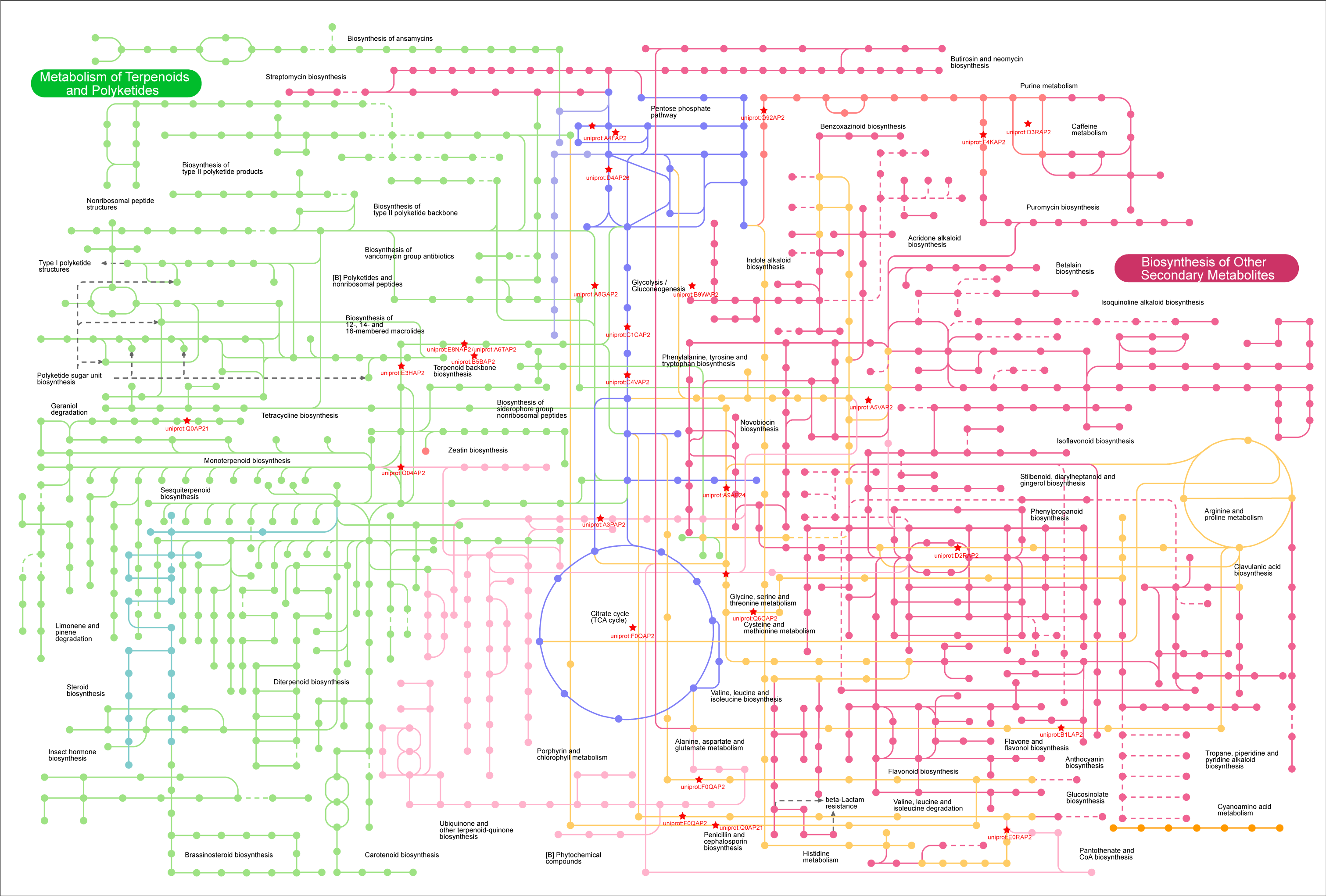

Supplement: Additional file 2: Figure S1 — Phylogenetic tree constructed from the neighbor-joining method using AP2 family transcription factor domains in Chinese cabbage and Arabidopsis. The numbers are bootstrap values based on 1000 iterations. Only bootstrap values larger than 50 are indicated. Figure S2. Phylogenetic tree constructed from the neighbor-joining method using AP2 family transcription factor domains in all 16 species analyzed. Figure S3. AP2/ERF protein motifs from each of the species examined. Figure S4. The ERF subfamily protein motifs derived from each species examined. Figure S5. The DREB subfamily protein motifs derived from each species. Figure S6. The RAV, AP2 and Soloist family protein motifs derived from each species examined. Figure S7. The AP2/ERF superfamily protein motifs derived from each species examined. Figure S8. Comparative analysis of synteny and expansion of AP2/ERF genes. Ten Chinese cabbage and five Arabidopsis chromosome maps were based on the orthologue pair positions, and demonstrate highly conserved synteny. Figure S9. Comparative analysis of synteny and expansion of AP2/ERF genes. Ten Chinese cabbage chromosome maps were based on the paralogue pair positions; and demonstrate highly conserved synteny. Figure S10. The secondary metabolic biosynthesis pathways of the AP2/ERF proteins. Figure S11. The regulatory pathways of the AP2/ERF proteins. Figure S12. The metabolic pathways of the AP2/ERF proteins. Figure S13. AP2/ERF transcription factors classification in Chinese cabbage. The size of each section is proportional to the relative abundance of the AP2/ERF genes assigned to the specific family. Figure S14. Distribution of AP2/ERF transcription factors in various Chinese cabbage tissues. Figure S15. Expression profile cluster analyses from Chinese cabbage DREB subfamily genes. Figure S16. Expression profile cluster analyses from Chinese cabbage RAV family genes. Figure S17. Expression profile cluster analyses from Chinese cabbage AP2 family genes. Figure S18. Chines [file 1471-2164-14-573-S2.zip › Figure S10.png]

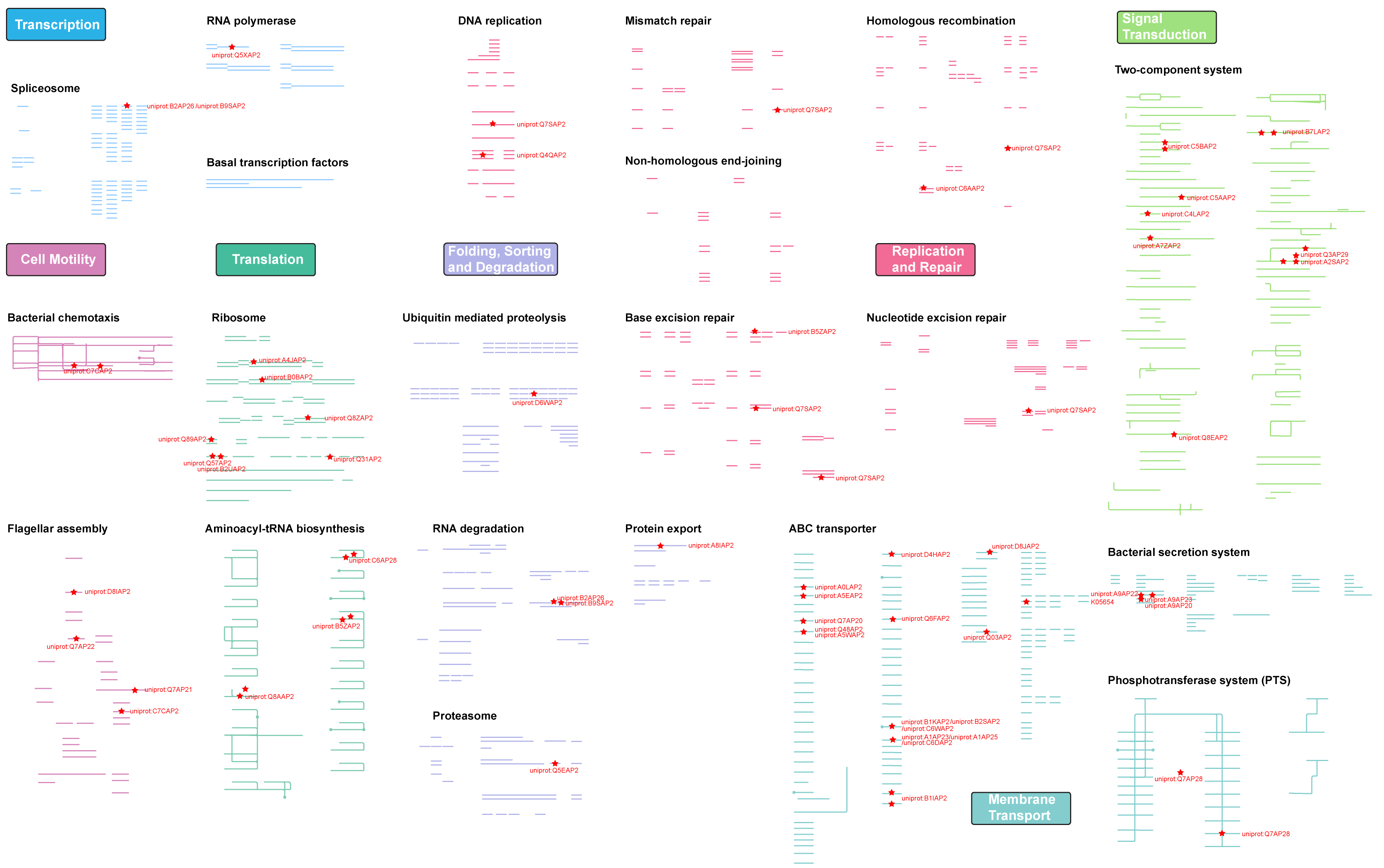

Supplement: Additional file 2: Figure S1 — Phylogenetic tree constructed from the neighbor-joining method using AP2 family transcription factor domains in Chinese cabbage and Arabidopsis. The numbers are bootstrap values based on 1000 iterations. Only bootstrap values larger than 50 are indicated. Figure S2. Phylogenetic tree constructed from the neighbor-joining method using AP2 family transcription factor domains in all 16 species analyzed. Figure S3. AP2/ERF protein motifs from each of the species examined. Figure S4. The ERF subfamily protein motifs derived from each species examined. Figure S5. The DREB subfamily protein motifs derived from each species. Figure S6. The RAV, AP2 and Soloist family protein motifs derived from each species examined. Figure S7. The AP2/ERF superfamily protein motifs derived from each species examined. Figure S8. Comparative analysis of synteny and expansion of AP2/ERF genes. Ten Chinese cabbage and five Arabidopsis chromosome maps were based on the orthologue pair positions, and demonstrate highly conserved synteny. Figure S9. Comparative analysis of synteny and expansion of AP2/ERF genes. Ten Chinese cabbage chromosome maps were based on the paralogue pair positions; and demonstrate highly conserved synteny. Figure S10. The secondary metabolic biosynthesis pathways of the AP2/ERF proteins. Figure S11. The regulatory pathways of the AP2/ERF proteins. Figure S12. The metabolic pathways of the AP2/ERF proteins. Figure S13. AP2/ERF transcription factors classification in Chinese cabbage. The size of each section is proportional to the relative abundance of the AP2/ERF genes assigned to the specific family. Figure S14. Distribution of AP2/ERF transcription factors in various Chinese cabbage tissues. Figure S15. Expression profile cluster analyses from Chinese cabbage DREB subfamily genes. Figure S16. Expression profile cluster analyses from Chinese cabbage RAV family genes. Figure S17. Expression profile cluster analyses from Chinese cabbage AP2 family genes. Figure S18. Chines [file 1471-2164-14-573-S2.zip › Figure S11.png]

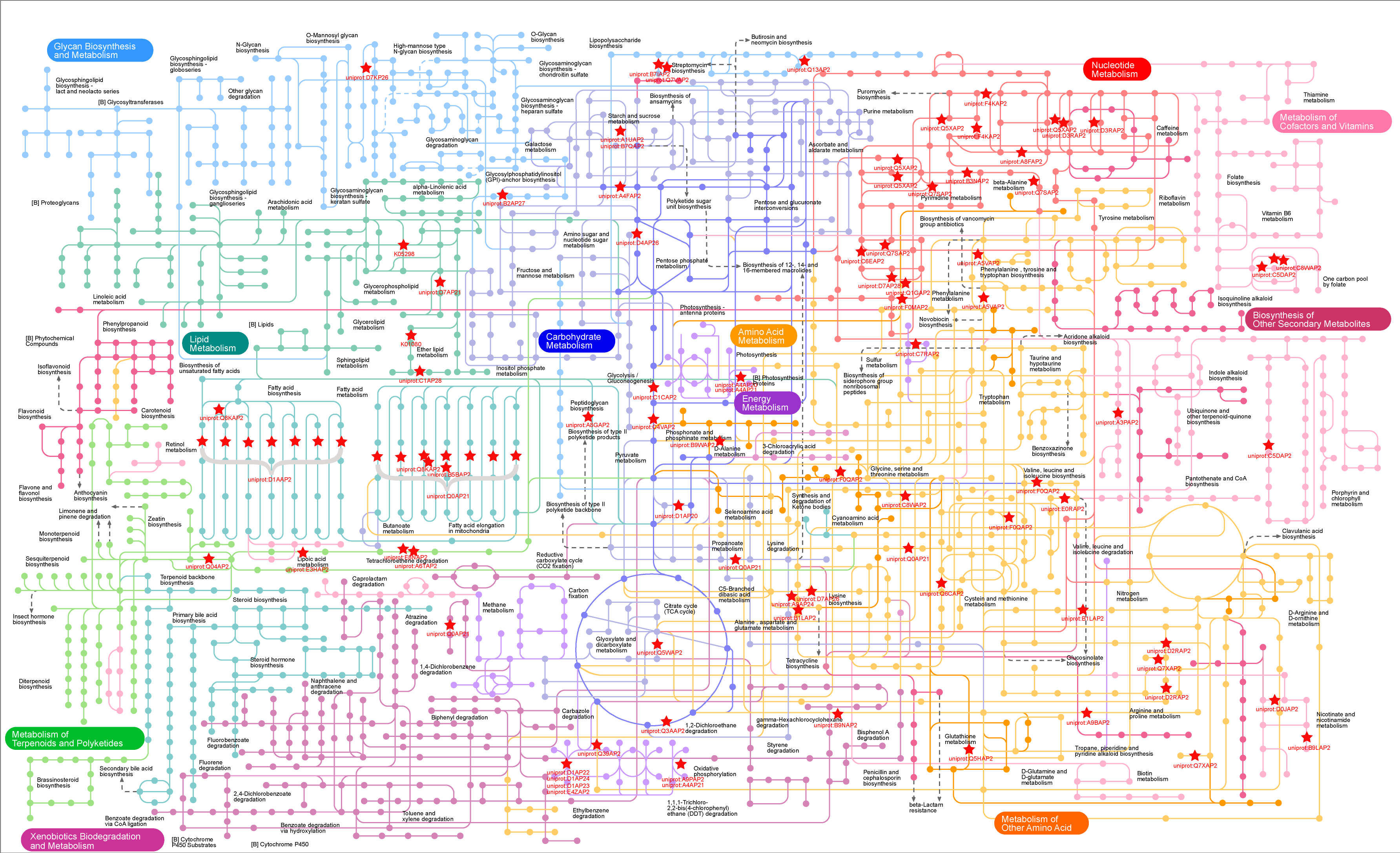

Supplement: Additional file 2: Figure S1 — Phylogenetic tree constructed from the neighbor-joining method using AP2 family transcription factor domains in Chinese cabbage and Arabidopsis. The numbers are bootstrap values based on 1000 iterations. Only bootstrap values larger than 50 are indicated. Figure S2. Phylogenetic tree constructed from the neighbor-joining method using AP2 family transcription factor domains in all 16 species analyzed. Figure S3. AP2/ERF protein motifs from each of the species examined. Figure S4. The ERF subfamily protein motifs derived from each species examined. Figure S5. The DREB subfamily protein motifs derived from each species. Figure S6. The RAV, AP2 and Soloist family protein motifs derived from each species examined. Figure S7. The AP2/ERF superfamily protein motifs derived from each species examined. Figure S8. Comparative analysis of synteny and expansion of AP2/ERF genes. Ten Chinese cabbage and five Arabidopsis chromosome maps were based on the orthologue pair positions, and demonstrate highly conserved synteny. Figure S9. Comparative analysis of synteny and expansion of AP2/ERF genes. Ten Chinese cabbage chromosome maps were based on the paralogue pair positions; and demonstrate highly conserved synteny. Figure S10. The secondary metabolic biosynthesis pathways of the AP2/ERF proteins. Figure S11. The regulatory pathways of the AP2/ERF proteins. Figure S12. The metabolic pathways of the AP2/ERF proteins. Figure S13. AP2/ERF transcription factors classification in Chinese cabbage. The size of each section is proportional to the relative abundance of the AP2/ERF genes assigned to the specific family. Figure S14. Distribution of AP2/ERF transcription factors in various Chinese cabbage tissues. Figure S15. Expression profile cluster analyses from Chinese cabbage DREB subfamily genes. Figure S16. Expression profile cluster analyses from Chinese cabbage RAV family genes. Figure S17. Expression profile cluster analyses from Chinese cabbage AP2 family genes. Figure S18. Chines [file 1471-2164-14-573-S2.zip › Figure S12.png]

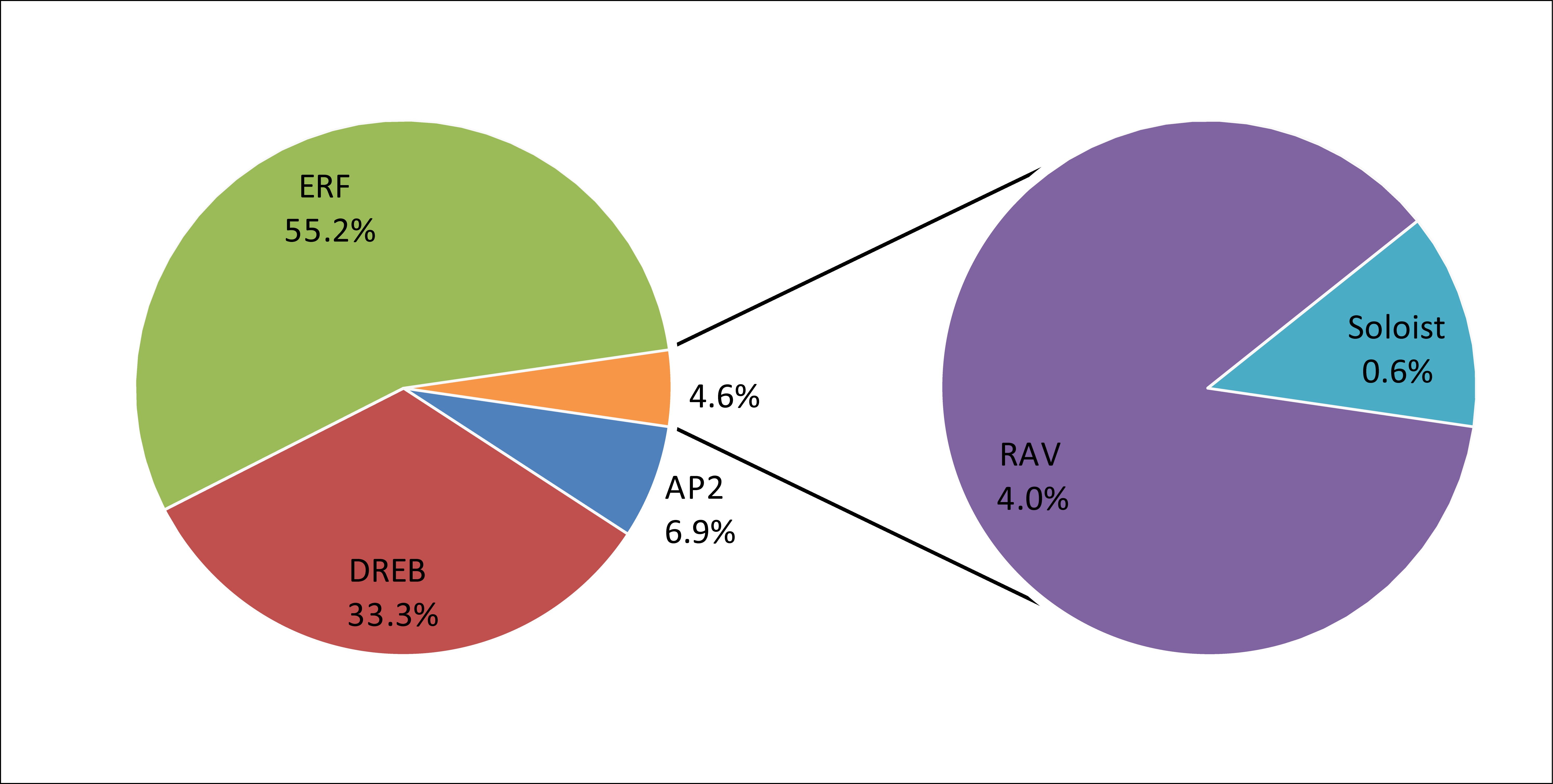

Supplement: Additional file 2: Figure S1 — Phylogenetic tree constructed from the neighbor-joining method using AP2 family transcription factor domains in Chinese cabbage and Arabidopsis. The numbers are bootstrap values based on 1000 iterations. Only bootstrap values larger than 50 are indicated. Figure S2. Phylogenetic tree constructed from the neighbor-joining method using AP2 family transcription factor domains in all 16 species analyzed. Figure S3. AP2/ERF protein motifs from each of the species examined. Figure S4. The ERF subfamily protein motifs derived from each species examined. Figure S5. The DREB subfamily protein motifs derived from each species. Figure S6. The RAV, AP2 and Soloist family protein motifs derived from each species examined. Figure S7. The AP2/ERF superfamily protein motifs derived from each species examined. Figure S8. Comparative analysis of synteny and expansion of AP2/ERF genes. Ten Chinese cabbage and five Arabidopsis chromosome maps were based on the orthologue pair positions, and demonstrate highly conserved synteny. Figure S9. Comparative analysis of synteny and expansion of AP2/ERF genes. Ten Chinese cabbage chromosome maps were based on the paralogue pair positions; and demonstrate highly conserved synteny. Figure S10. The secondary metabolic biosynthesis pathways of the AP2/ERF proteins. Figure S11. The regulatory pathways of the AP2/ERF proteins. Figure S12. The metabolic pathways of the AP2/ERF proteins. Figure S13. AP2/ERF transcription factors classification in Chinese cabbage. The size of each section is proportional to the relative abundance of the AP2/ERF genes assigned to the specific family. Figure S14. Distribution of AP2/ERF transcription factors in various Chinese cabbage tissues. Figure S15. Expression profile cluster analyses from Chinese cabbage DREB subfamily genes. Figure S16. Expression profile cluster analyses from Chinese cabbage RAV family genes. Figure S17. Expression profile cluster analyses from Chinese cabbage AP2 family genes. Figure S18. Chines [file 1471-2164-14-573-S2.zip › Figure S13.png]

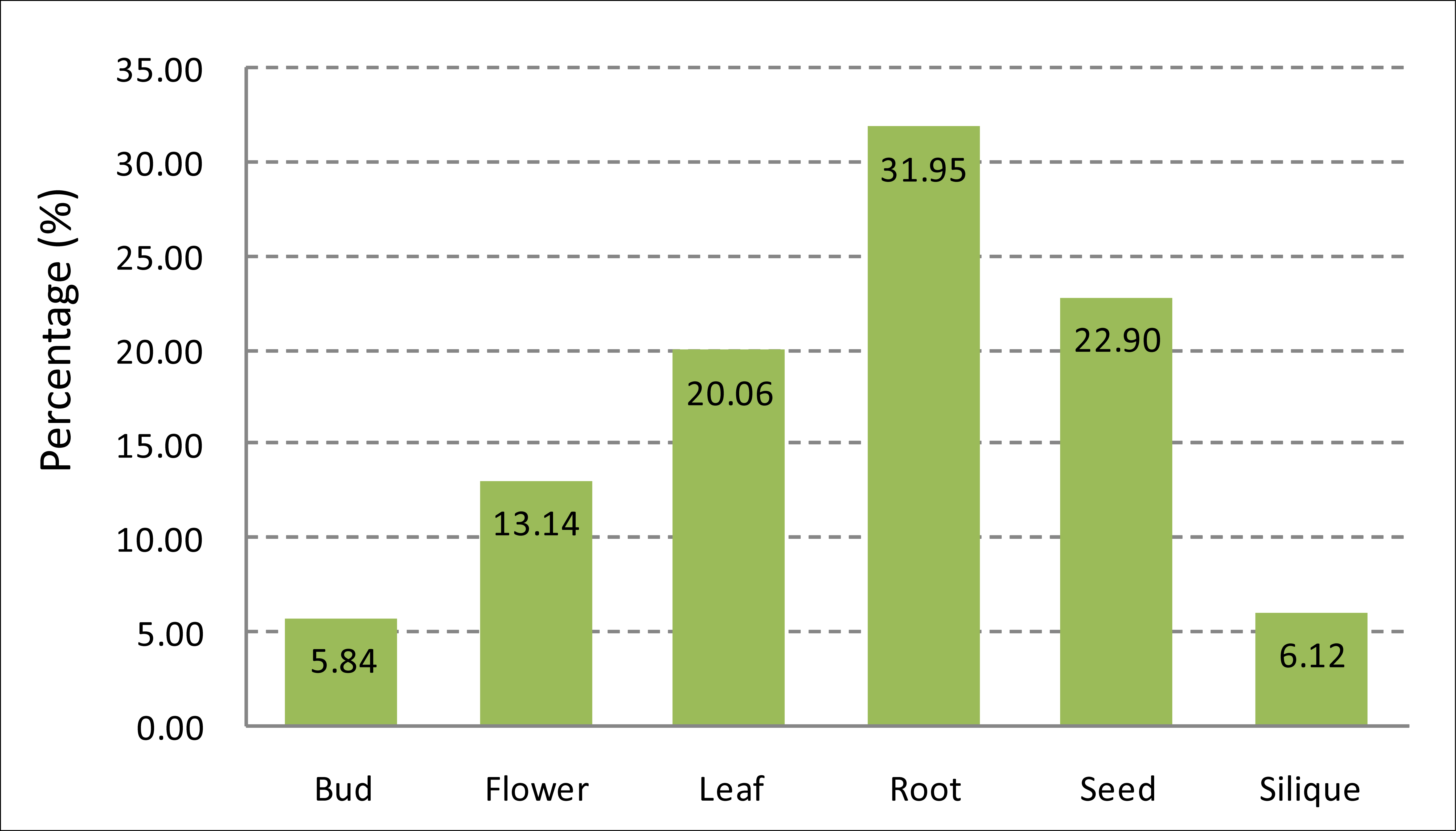

Supplement: Additional file 2: Figure S1 — Phylogenetic tree constructed from the neighbor-joining method using AP2 family transcription factor domains in Chinese cabbage and Arabidopsis. The numbers are bootstrap values based on 1000 iterations. Only bootstrap values larger than 50 are indicated. Figure S2. Phylogenetic tree constructed from the neighbor-joining method using AP2 family transcription factor domains in all 16 species analyzed. Figure S3. AP2/ERF protein motifs from each of the species examined. Figure S4. The ERF subfamily protein motifs derived from each species examined. Figure S5. The DREB subfamily protein motifs derived from each species. Figure S6. The RAV, AP2 and Soloist family protein motifs derived from each species examined. Figure S7. The AP2/ERF superfamily protein motifs derived from each species examined. Figure S8. Comparative analysis of synteny and expansion of AP2/ERF genes. Ten Chinese cabbage and five Arabidopsis chromosome maps were based on the orthologue pair positions, and demonstrate highly conserved synteny. Figure S9. Comparative analysis of synteny and expansion of AP2/ERF genes. Ten Chinese cabbage chromosome maps were based on the paralogue pair positions; and demonstrate highly conserved synteny. Figure S10. The secondary metabolic biosynthesis pathways of the AP2/ERF proteins. Figure S11. The regulatory pathways of the AP2/ERF proteins. Figure S12. The metabolic pathways of the AP2/ERF proteins. Figure S13. AP2/ERF transcription factors classification in Chinese cabbage. The size of each section is proportional to the relative abundance of the AP2/ERF genes assigned to the specific family. Figure S14. Distribution of AP2/ERF transcription factors in various Chinese cabbage tissues. Figure S15. Expression profile cluster analyses from Chinese cabbage DREB subfamily genes. Figure S16. Expression profile cluster analyses from Chinese cabbage RAV family genes. Figure S17. Expression profile cluster analyses from Chinese cabbage AP2 family genes. Figure S18. Chines [file 1471-2164-14-573-S2.zip › Figure S14.png]

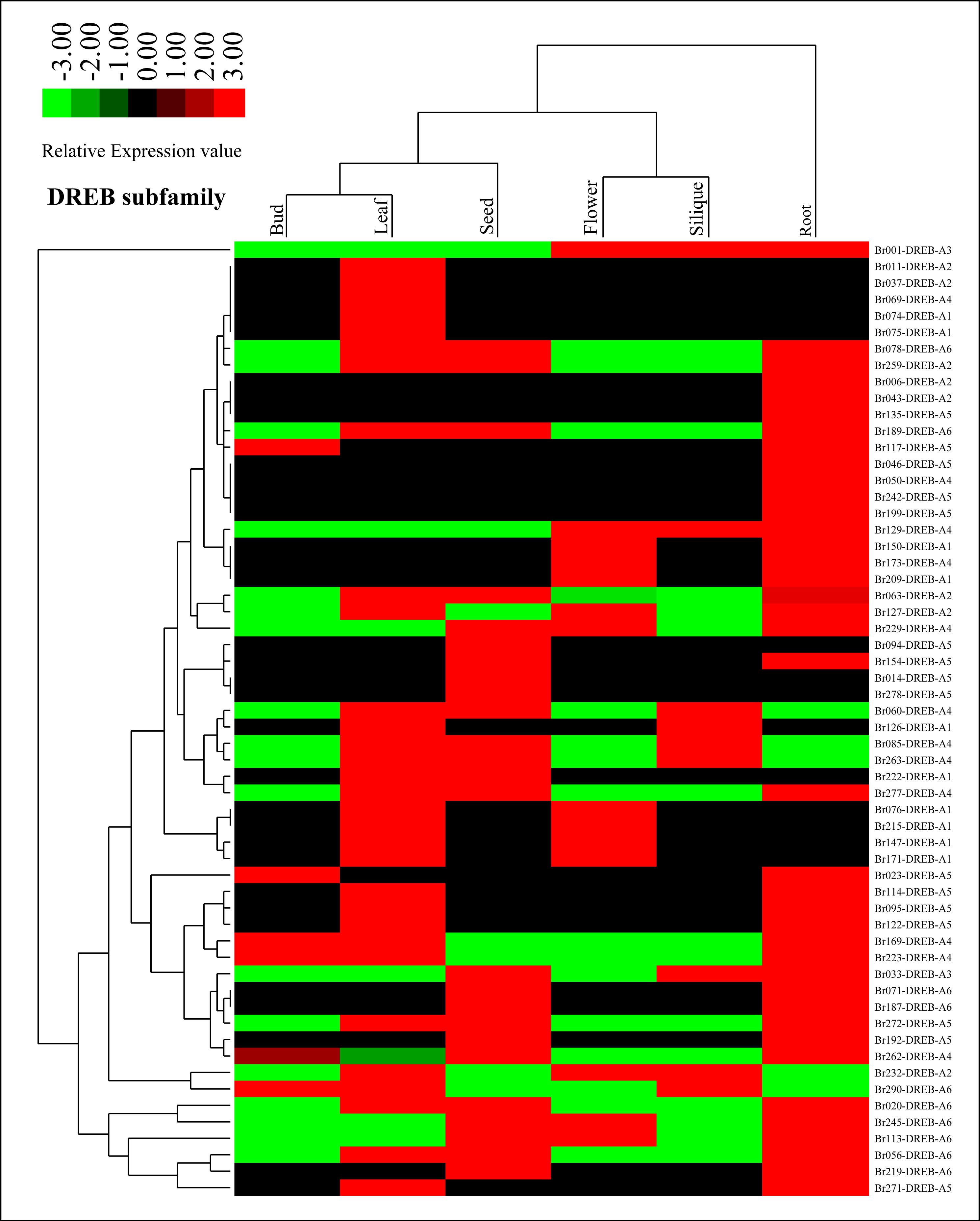

Supplement: Additional file 2: Figure S1 — Phylogenetic tree constructed from the neighbor-joining method using AP2 family transcription factor domains in Chinese cabbage and Arabidopsis. The numbers are bootstrap values based on 1000 iterations. Only bootstrap values larger than 50 are indicated. Figure S2. Phylogenetic tree constructed from the neighbor-joining method using AP2 family transcription factor domains in all 16 species analyzed. Figure S3. AP2/ERF protein motifs from each of the species examined. Figure S4. The ERF subfamily protein motifs derived from each species examined. Figure S5. The DREB subfamily protein motifs derived from each species. Figure S6. The RAV, AP2 and Soloist family protein motifs derived from each species examined. Figure S7. The AP2/ERF superfamily protein motifs derived from each species examined. Figure S8. Comparative analysis of synteny and expansion of AP2/ERF genes. Ten Chinese cabbage and five Arabidopsis chromosome maps were based on the orthologue pair positions, and demonstrate highly conserved synteny. Figure S9. Comparative analysis of synteny and expansion of AP2/ERF genes. Ten Chinese cabbage chromosome maps were based on the paralogue pair positions; and demonstrate highly conserved synteny. Figure S10. The secondary metabolic biosynthesis pathways of the AP2/ERF proteins. Figure S11. The regulatory pathways of the AP2/ERF proteins. Figure S12. The metabolic pathways of the AP2/ERF proteins. Figure S13. AP2/ERF transcription factors classification in Chinese cabbage. The size of each section is proportional to the relative abundance of the AP2/ERF genes assigned to the specific family. Figure S14. Distribution of AP2/ERF transcription factors in various Chinese cabbage tissues. Figure S15. Expression profile cluster analyses from Chinese cabbage DREB subfamily genes. Figure S16. Expression profile cluster analyses from Chinese cabbage RAV family genes. Figure S17. Expression profile cluster analyses from Chinese cabbage AP2 family genes. Figure S18. Chines [file 1471-2164-14-573-S2.zip › Figure S15.png]

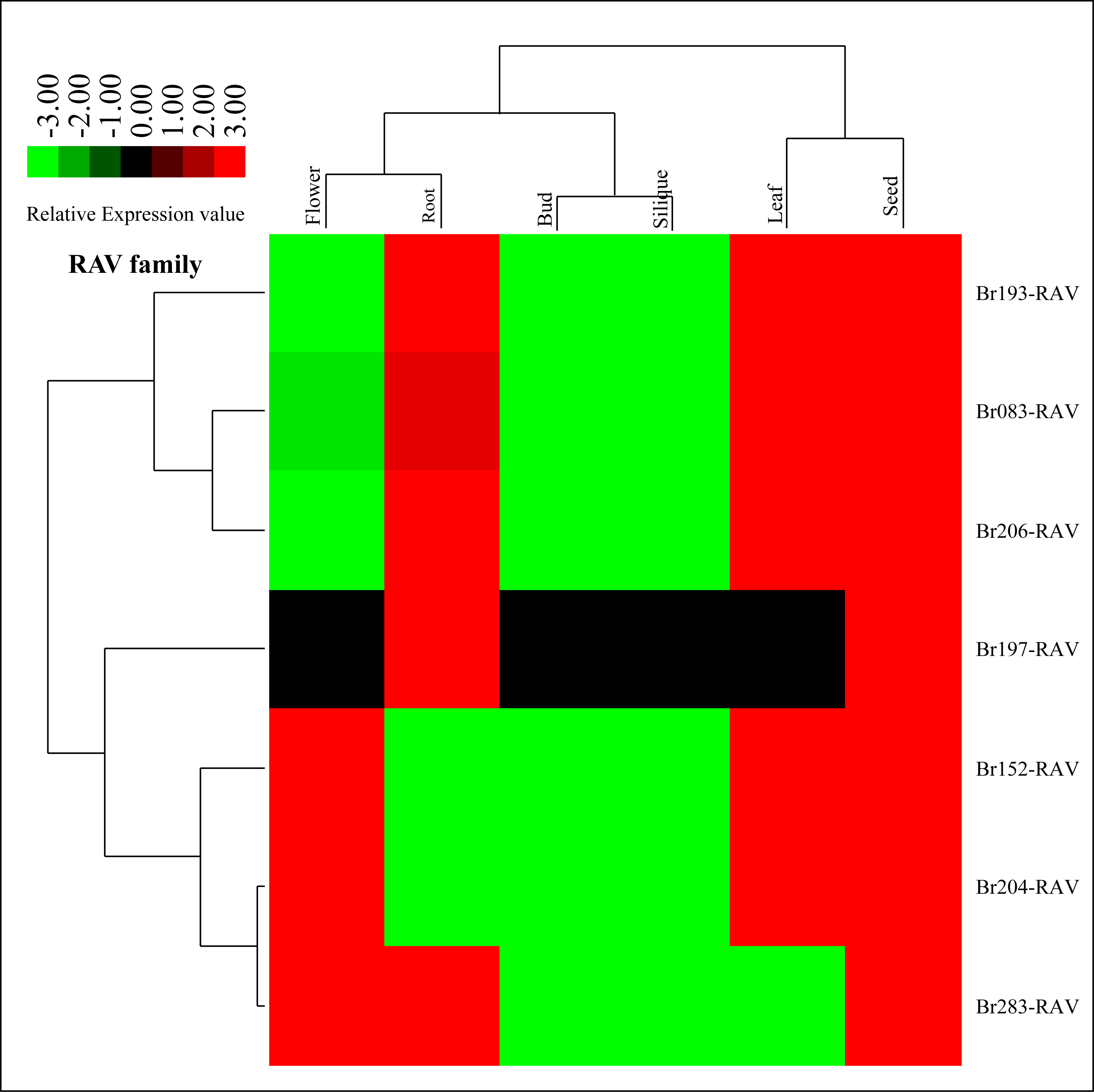

Supplement: Additional file 2: Figure S1 — Phylogenetic tree constructed from the neighbor-joining method using AP2 family transcription factor domains in Chinese cabbage and Arabidopsis. The numbers are bootstrap values based on 1000 iterations. Only bootstrap values larger than 50 are indicated. Figure S2. Phylogenetic tree constructed from the neighbor-joining method using AP2 family transcription factor domains in all 16 species analyzed. Figure S3. AP2/ERF protein motifs from each of the species examined. Figure S4. The ERF subfamily protein motifs derived from each species examined. Figure S5. The DREB subfamily protein motifs derived from each species. Figure S6. The RAV, AP2 and Soloist family protein motifs derived from each species examined. Figure S7. The AP2/ERF superfamily protein motifs derived from each species examined. Figure S8. Comparative analysis of synteny and expansion of AP2/ERF genes. Ten Chinese cabbage and five Arabidopsis chromosome maps were based on the orthologue pair positions, and demonstrate highly conserved synteny. Figure S9. Comparative analysis of synteny and expansion of AP2/ERF genes. Ten Chinese cabbage chromosome maps were based on the paralogue pair positions; and demonstrate highly conserved synteny. Figure S10. The secondary metabolic biosynthesis pathways of the AP2/ERF proteins. Figure S11. The regulatory pathways of the AP2/ERF proteins. Figure S12. The metabolic pathways of the AP2/ERF proteins. Figure S13. AP2/ERF transcription factors classification in Chinese cabbage. The size of each section is proportional to the relative abundance of the AP2/ERF genes assigned to the specific family. Figure S14. Distribution of AP2/ERF transcription factors in various Chinese cabbage tissues. Figure S15. Expression profile cluster analyses from Chinese cabbage DREB subfamily genes. Figure S16. Expression profile cluster analyses from Chinese cabbage RAV family genes. Figure S17. Expression profile cluster analyses from Chinese cabbage AP2 family genes. Figure S18. Chines [file 1471-2164-14-573-S2.zip › Figure S16.png]

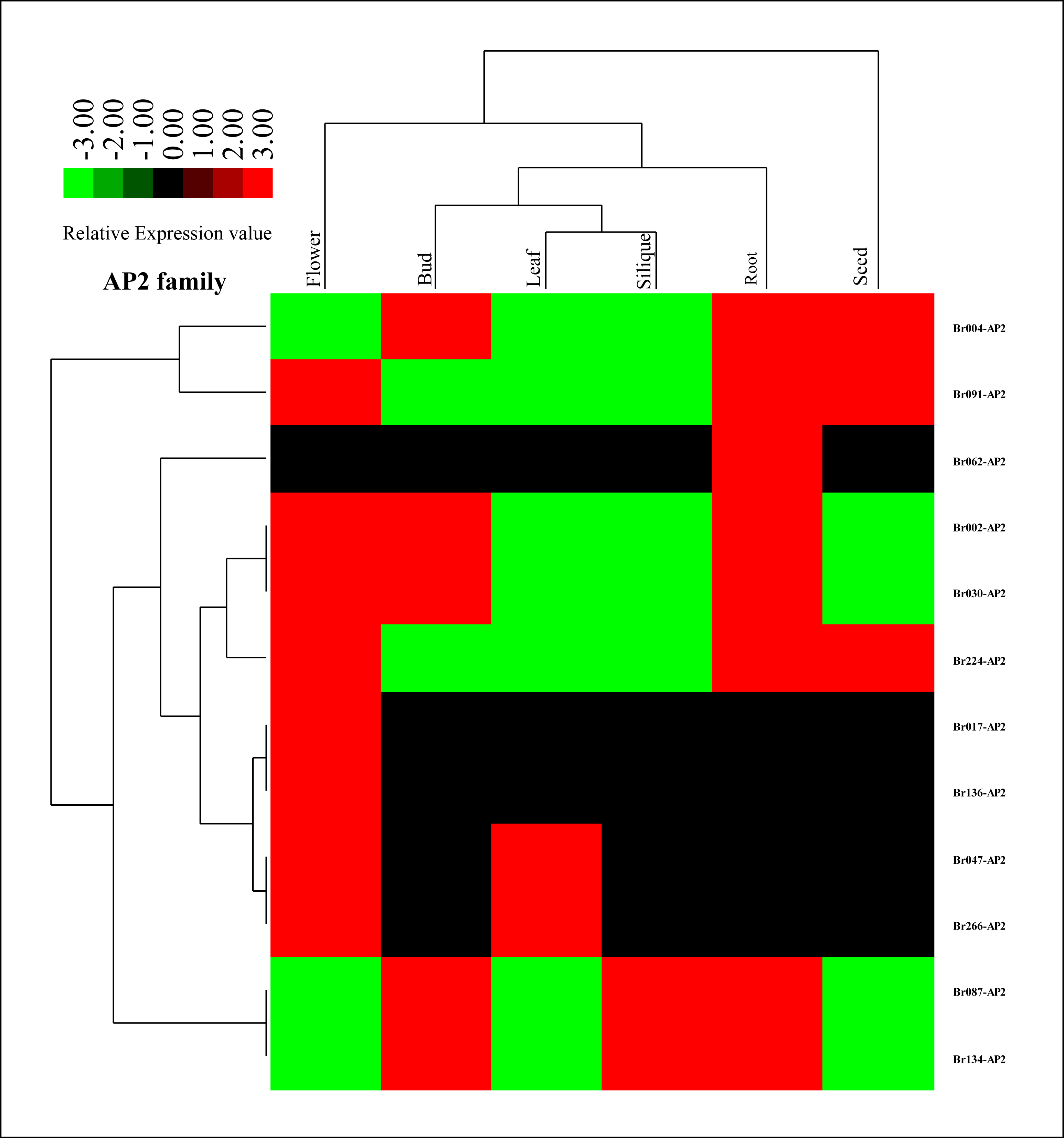

Supplement: Additional file 2: Figure S1 — Phylogenetic tree constructed from the neighbor-joining method using AP2 family transcription factor domains in Chinese cabbage and Arabidopsis. The numbers are bootstrap values based on 1000 iterations. Only bootstrap values larger than 50 are indicated. Figure S2. Phylogenetic tree constructed from the neighbor-joining method using AP2 family transcription factor domains in all 16 species analyzed. Figure S3. AP2/ERF protein motifs from each of the species examined. Figure S4. The ERF subfamily protein motifs derived from each species examined. Figure S5. The DREB subfamily protein motifs derived from each species. Figure S6. The RAV, AP2 and Soloist family protein motifs derived from each species examined. Figure S7. The AP2/ERF superfamily protein motifs derived from each species examined. Figure S8. Comparative analysis of synteny and expansion of AP2/ERF genes. Ten Chinese cabbage and five Arabidopsis chromosome maps were based on the orthologue pair positions, and demonstrate highly conserved synteny. Figure S9. Comparative analysis of synteny and expansion of AP2/ERF genes. Ten Chinese cabbage chromosome maps were based on the paralogue pair positions; and demonstrate highly conserved synteny. Figure S10. The secondary metabolic biosynthesis pathways of the AP2/ERF proteins. Figure S11. The regulatory pathways of the AP2/ERF proteins. Figure S12. The metabolic pathways of the AP2/ERF proteins. Figure S13. AP2/ERF transcription factors classification in Chinese cabbage. The size of each section is proportional to the relative abundance of the AP2/ERF genes assigned to the specific family. Figure S14. Distribution of AP2/ERF transcription factors in various Chinese cabbage tissues. Figure S15. Expression profile cluster analyses from Chinese cabbage DREB subfamily genes. Figure S16. Expression profile cluster analyses from Chinese cabbage RAV family genes. Figure S17. Expression profile cluster analyses from Chinese cabbage AP2 family genes. Figure S18. Chines [file 1471-2164-14-573-S2.zip › Figure S17.png]

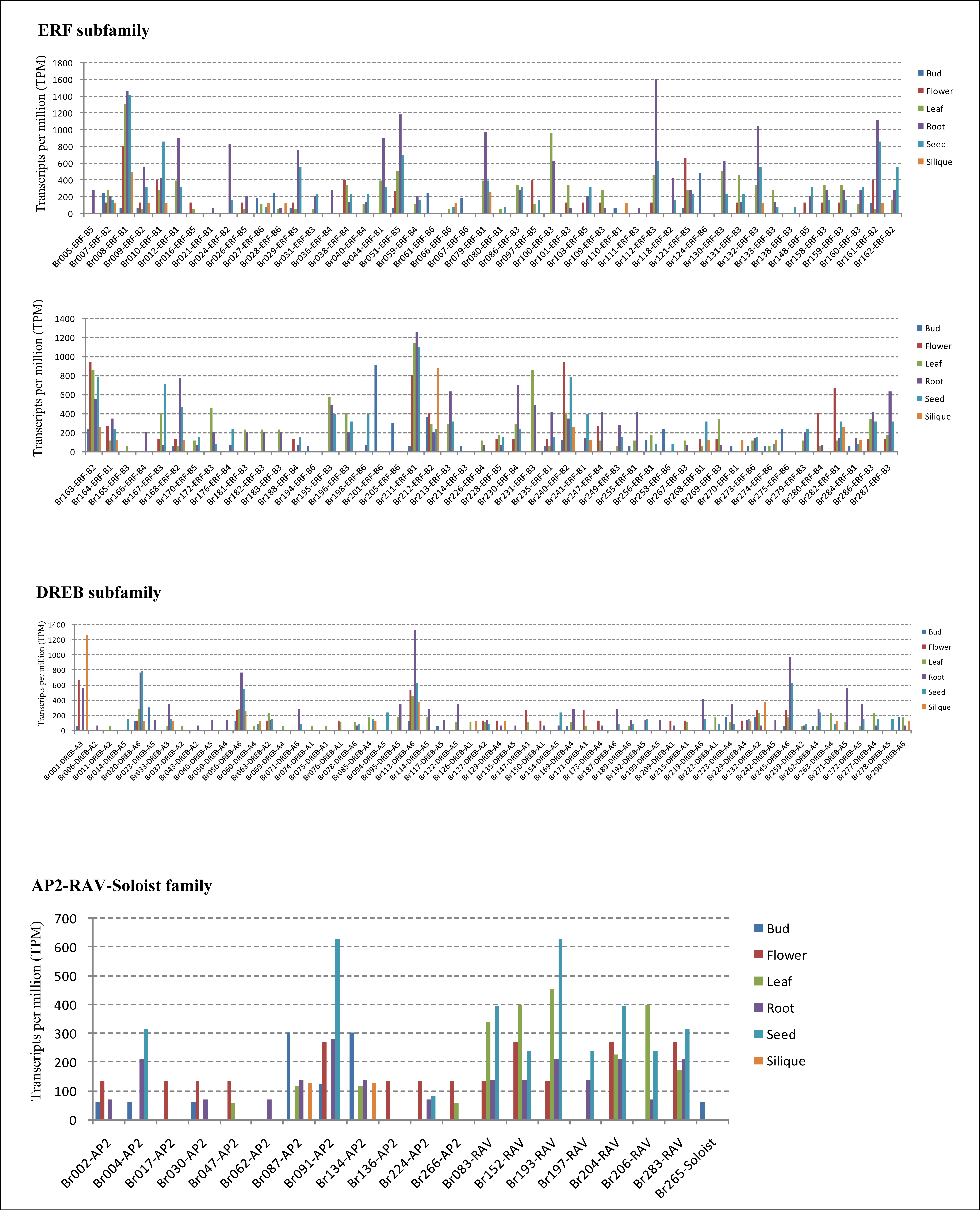

Supplement: Additional file 2: Figure S1 — Phylogenetic tree constructed from the neighbor-joining method using AP2 family transcription factor domains in Chinese cabbage and Arabidopsis. The numbers are bootstrap values based on 1000 iterations. Only bootstrap values larger than 50 are indicated. Figure S2. Phylogenetic tree constructed from the neighbor-joining method using AP2 family transcription factor domains in all 16 species analyzed. Figure S3. AP2/ERF protein motifs from each of the species examined. Figure S4. The ERF subfamily protein motifs derived from each species examined. Figure S5. The DREB subfamily protein motifs derived from each species. Figure S6. The RAV, AP2 and Soloist family protein motifs derived from each species examined. Figure S7. The AP2/ERF superfamily protein motifs derived from each species examined. Figure S8. Comparative analysis of synteny and expansion of AP2/ERF genes. Ten Chinese cabbage and five Arabidopsis chromosome maps were based on the orthologue pair positions, and demonstrate highly conserved synteny. Figure S9. Comparative analysis of synteny and expansion of AP2/ERF genes. Ten Chinese cabbage chromosome maps were based on the paralogue pair positions; and demonstrate highly conserved synteny. Figure S10. The secondary metabolic biosynthesis pathways of the AP2/ERF proteins. Figure S11. The regulatory pathways of the AP2/ERF proteins. Figure S12. The metabolic pathways of the AP2/ERF proteins. Figure S13. AP2/ERF transcription factors classification in Chinese cabbage. The size of each section is proportional to the relative abundance of the AP2/ERF genes assigned to the specific family. Figure S14. Distribution of AP2/ERF transcription factors in various Chinese cabbage tissues. Figure S15. Expression profile cluster analyses from Chinese cabbage DREB subfamily genes. Figure S16. Expression profile cluster analyses from Chinese cabbage RAV family genes. Figure S17. Expression profile cluster analyses from Chinese cabbage AP2 family genes. Figure S18. Chines [file 1471-2164-14-573-S2.zip › Figure S18.png]

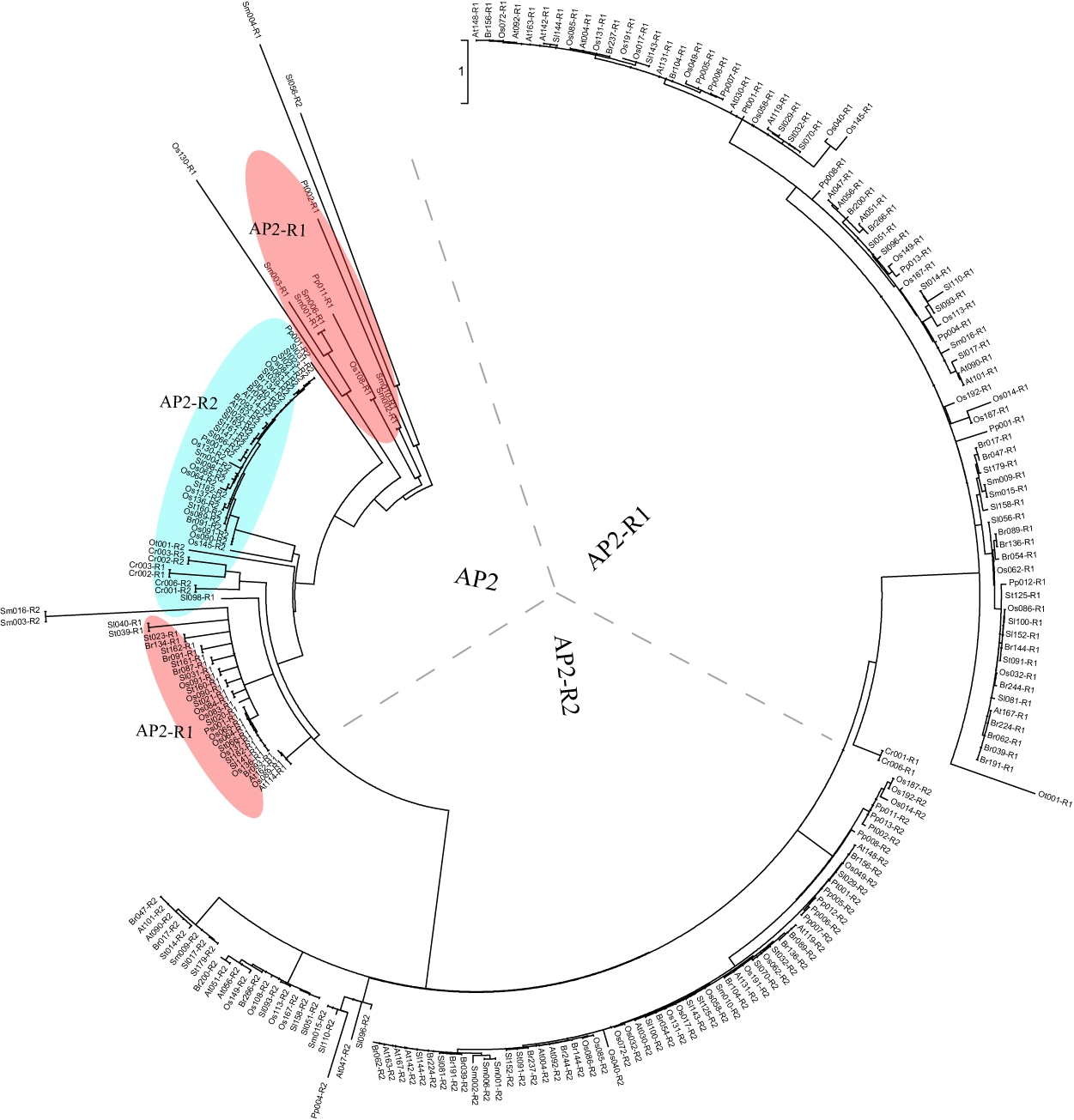

Supplement: Additional file 2: Figure S1 — Phylogenetic tree constructed from the neighbor-joining method using AP2 family transcription factor domains in Chinese cabbage and Arabidopsis. The numbers are bootstrap values based on 1000 iterations. Only bootstrap values larger than 50 are indicated. Figure S2. Phylogenetic tree constructed from the neighbor-joining method using AP2 family transcription factor domains in all 16 species analyzed. Figure S3. AP2/ERF protein motifs from each of the species examined. Figure S4. The ERF subfamily protein motifs derived from each species examined. Figure S5. The DREB subfamily protein motifs derived from each species. Figure S6. The RAV, AP2 and Soloist family protein motifs derived from each species examined. Figure S7. The AP2/ERF superfamily protein motifs derived from each species examined. Figure S8. Comparative analysis of synteny and expansion of AP2/ERF genes. Ten Chinese cabbage and five Arabidopsis chromosome maps were based on the orthologue pair positions, and demonstrate highly conserved synteny. Figure S9. Comparative analysis of synteny and expansion of AP2/ERF genes. Ten Chinese cabbage chromosome maps were based on the paralogue pair positions; and demonstrate highly conserved synteny. Figure S10. The secondary metabolic biosynthesis pathways of the AP2/ERF proteins. Figure S11. The regulatory pathways of the AP2/ERF proteins. Figure S12. The metabolic pathways of the AP2/ERF proteins. Figure S13. AP2/ERF transcription factors classification in Chinese cabbage. The size of each section is proportional to the relative abundance of the AP2/ERF genes assigned to the specific family. Figure S14. Distribution of AP2/ERF transcription factors in various Chinese cabbage tissues. Figure S15. Expression profile cluster analyses from Chinese cabbage DREB subfamily genes. Figure S16. Expression profile cluster analyses from Chinese cabbage RAV family genes. Figure S17. Expression profile cluster analyses from Chinese cabbage AP2 family genes. Figure S18. Chines [file 1471-2164-14-573-S2.zip › Figure S2.png]

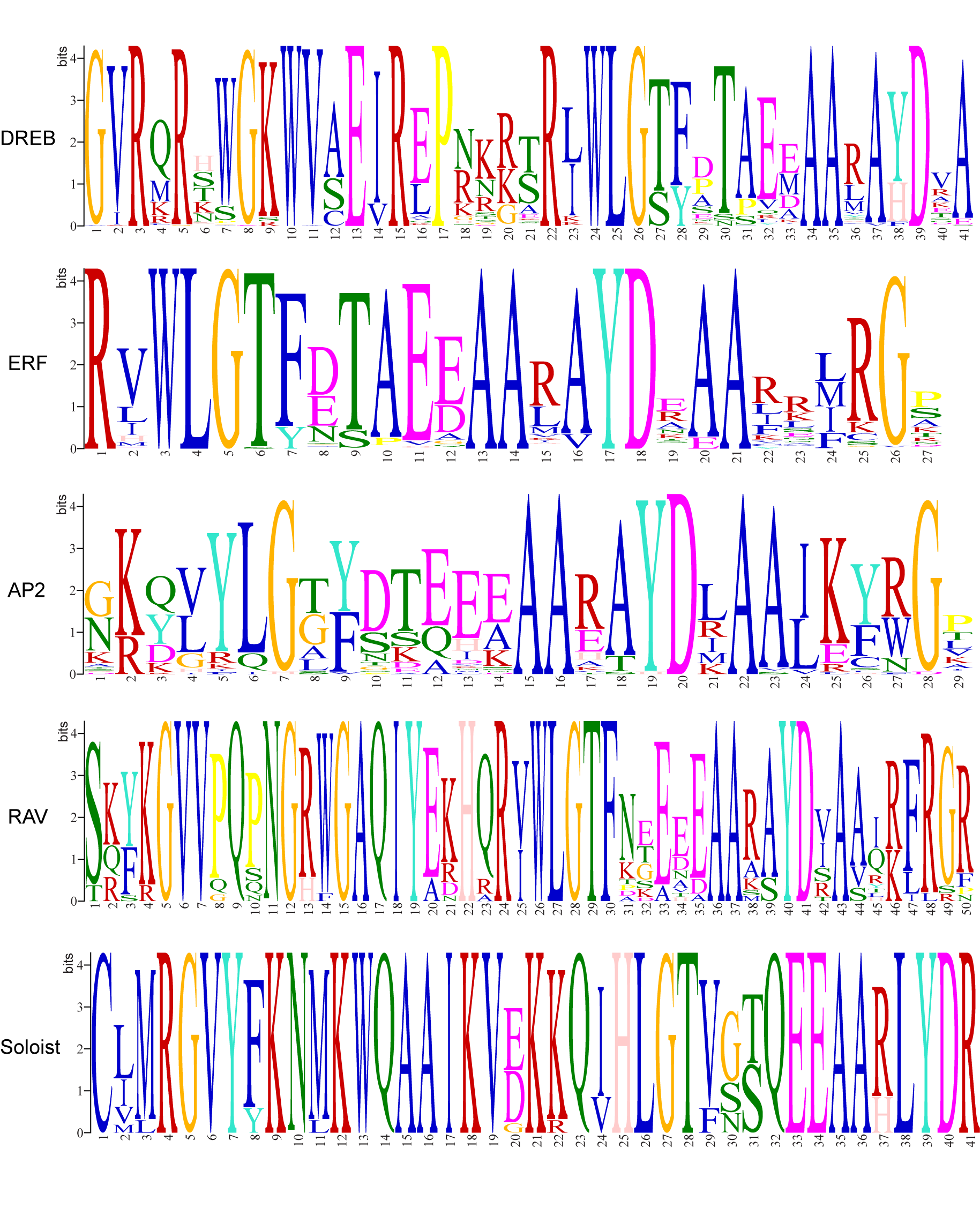

Supplement: Additional file 2: Figure S1 — Phylogenetic tree constructed from the neighbor-joining method using AP2 family transcription factor domains in Chinese cabbage and Arabidopsis. The numbers are bootstrap values based on 1000 iterations. Only bootstrap values larger than 50 are indicated. Figure S2. Phylogenetic tree constructed from the neighbor-joining method using AP2 family transcription factor domains in all 16 species analyzed. Figure S3. AP2/ERF protein motifs from each of the species examined. Figure S4. The ERF subfamily protein motifs derived from each species examined. Figure S5. The DREB subfamily protein motifs derived from each species. Figure S6. The RAV, AP2 and Soloist family protein motifs derived from each species examined. Figure S7. The AP2/ERF superfamily protein motifs derived from each species examined. Figure S8. Comparative analysis of synteny and expansion of AP2/ERF genes. Ten Chinese cabbage and five Arabidopsis chromosome maps were based on the orthologue pair positions, and demonstrate highly conserved synteny. Figure S9. Comparative analysis of synteny and expansion of AP2/ERF genes. Ten Chinese cabbage chromosome maps were based on the paralogue pair positions; and demonstrate highly conserved synteny. Figure S10. The secondary metabolic biosynthesis pathways of the AP2/ERF proteins. Figure S11. The regulatory pathways of the AP2/ERF proteins. Figure S12. The metabolic pathways of the AP2/ERF proteins. Figure S13. AP2/ERF transcription factors classification in Chinese cabbage. The size of each section is proportional to the relative abundance of the AP2/ERF genes assigned to the specific family. Figure S14. Distribution of AP2/ERF transcription factors in various Chinese cabbage tissues. Figure S15. Expression profile cluster analyses from Chinese cabbage DREB subfamily genes. Figure S16. Expression profile cluster analyses from Chinese cabbage RAV family genes. Figure S17. Expression profile cluster analyses from Chinese cabbage AP2 family genes. Figure S18. Chines [file 1471-2164-14-573-S2.zip › Figure S3.png]

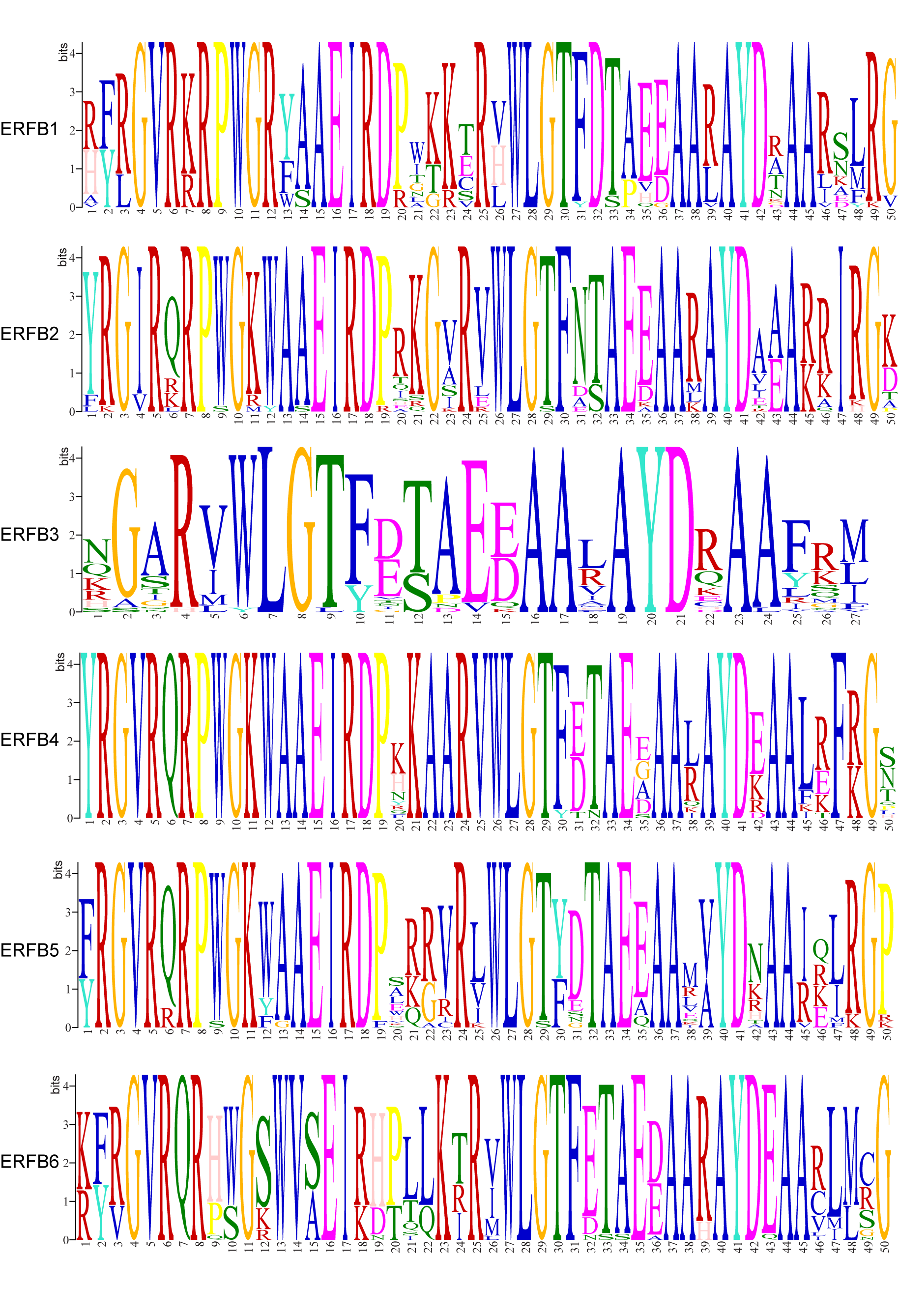

Supplement: Additional file 2: Figure S1 — Phylogenetic tree constructed from the neighbor-joining method using AP2 family transcription factor domains in Chinese cabbage and Arabidopsis. The numbers are bootstrap values based on 1000 iterations. Only bootstrap values larger than 50 are indicated. Figure S2. Phylogenetic tree constructed from the neighbor-joining method using AP2 family transcription factor domains in all 16 species analyzed. Figure S3. AP2/ERF protein motifs from each of the species examined. Figure S4. The ERF subfamily protein motifs derived from each species examined. Figure S5. The DREB subfamily protein motifs derived from each species. Figure S6. The RAV, AP2 and Soloist family protein motifs derived from each species examined. Figure S7. The AP2/ERF superfamily protein motifs derived from each species examined. Figure S8. Comparative analysis of synteny and expansion of AP2/ERF genes. Ten Chinese cabbage and five Arabidopsis chromosome maps were based on the orthologue pair positions, and demonstrate highly conserved synteny. Figure S9. Comparative analysis of synteny and expansion of AP2/ERF genes. Ten Chinese cabbage chromosome maps were based on the paralogue pair positions; and demonstrate highly conserved synteny. Figure S10. The secondary metabolic biosynthesis pathways of the AP2/ERF proteins. Figure S11. The regulatory pathways of the AP2/ERF proteins. Figure S12. The metabolic pathways of the AP2/ERF proteins. Figure S13. AP2/ERF transcription factors classification in Chinese cabbage. The size of each section is proportional to the relative abundance of the AP2/ERF genes assigned to the specific family. Figure S14. Distribution of AP2/ERF transcription factors in various Chinese cabbage tissues. Figure S15. Expression profile cluster analyses from Chinese cabbage DREB subfamily genes. Figure S16. Expression profile cluster analyses from Chinese cabbage RAV family genes. Figure S17. Expression profile cluster analyses from Chinese cabbage AP2 family genes. Figure S18. Chines [file 1471-2164-14-573-S2.zip › Figure S4.png]

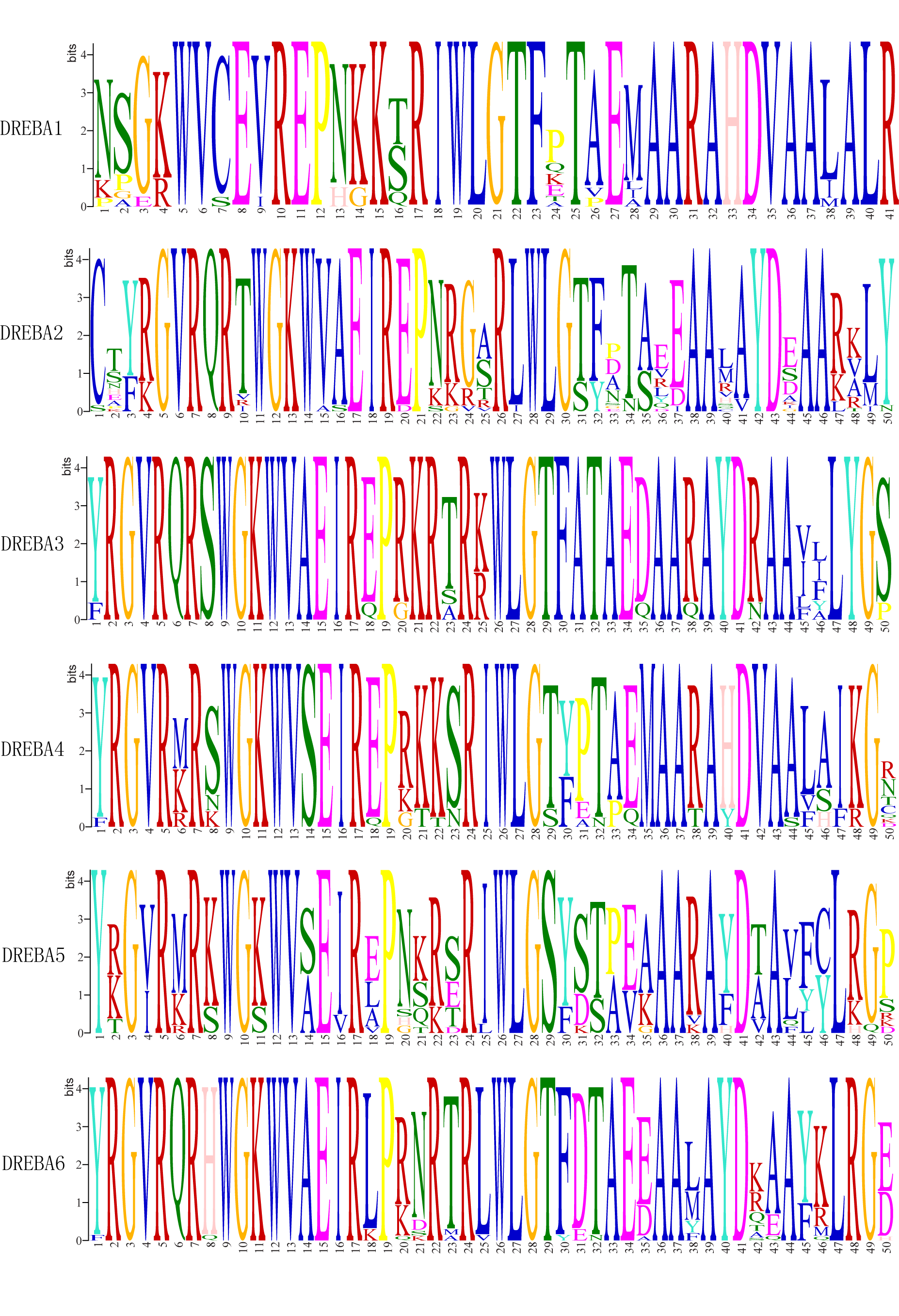

Supplement: Additional file 2: Figure S1 — Phylogenetic tree constructed from the neighbor-joining method using AP2 family transcription factor domains in Chinese cabbage and Arabidopsis. The numbers are bootstrap values based on 1000 iterations. Only bootstrap values larger than 50 are indicated. Figure S2. Phylogenetic tree constructed from the neighbor-joining method using AP2 family transcription factor domains in all 16 species analyzed. Figure S3. AP2/ERF protein motifs from each of the species examined. Figure S4. The ERF subfamily protein motifs derived from each species examined. Figure S5. The DREB subfamily protein motifs derived from each species. Figure S6. The RAV, AP2 and Soloist family protein motifs derived from each species examined. Figure S7. The AP2/ERF superfamily protein motifs derived from each species examined. Figure S8. Comparative analysis of synteny and expansion of AP2/ERF genes. Ten Chinese cabbage and five Arabidopsis chromosome maps were based on the orthologue pair positions, and demonstrate highly conserved synteny. Figure S9. Comparative analysis of synteny and expansion of AP2/ERF genes. Ten Chinese cabbage chromosome maps were based on the paralogue pair positions; and demonstrate highly conserved synteny. Figure S10. The secondary metabolic biosynthesis pathways of the AP2/ERF proteins. Figure S11. The regulatory pathways of the AP2/ERF proteins. Figure S12. The metabolic pathways of the AP2/ERF proteins. Figure S13. AP2/ERF transcription factors classification in Chinese cabbage. The size of each section is proportional to the relative abundance of the AP2/ERF genes assigned to the specific family. Figure S14. Distribution of AP2/ERF transcription factors in various Chinese cabbage tissues. Figure S15. Expression profile cluster analyses from Chinese cabbage DREB subfamily genes. Figure S16. Expression profile cluster analyses from Chinese cabbage RAV family genes. Figure S17. Expression profile cluster analyses from Chinese cabbage AP2 family genes. Figure S18. Chines [file 1471-2164-14-573-S2.zip › Figure S5.png]

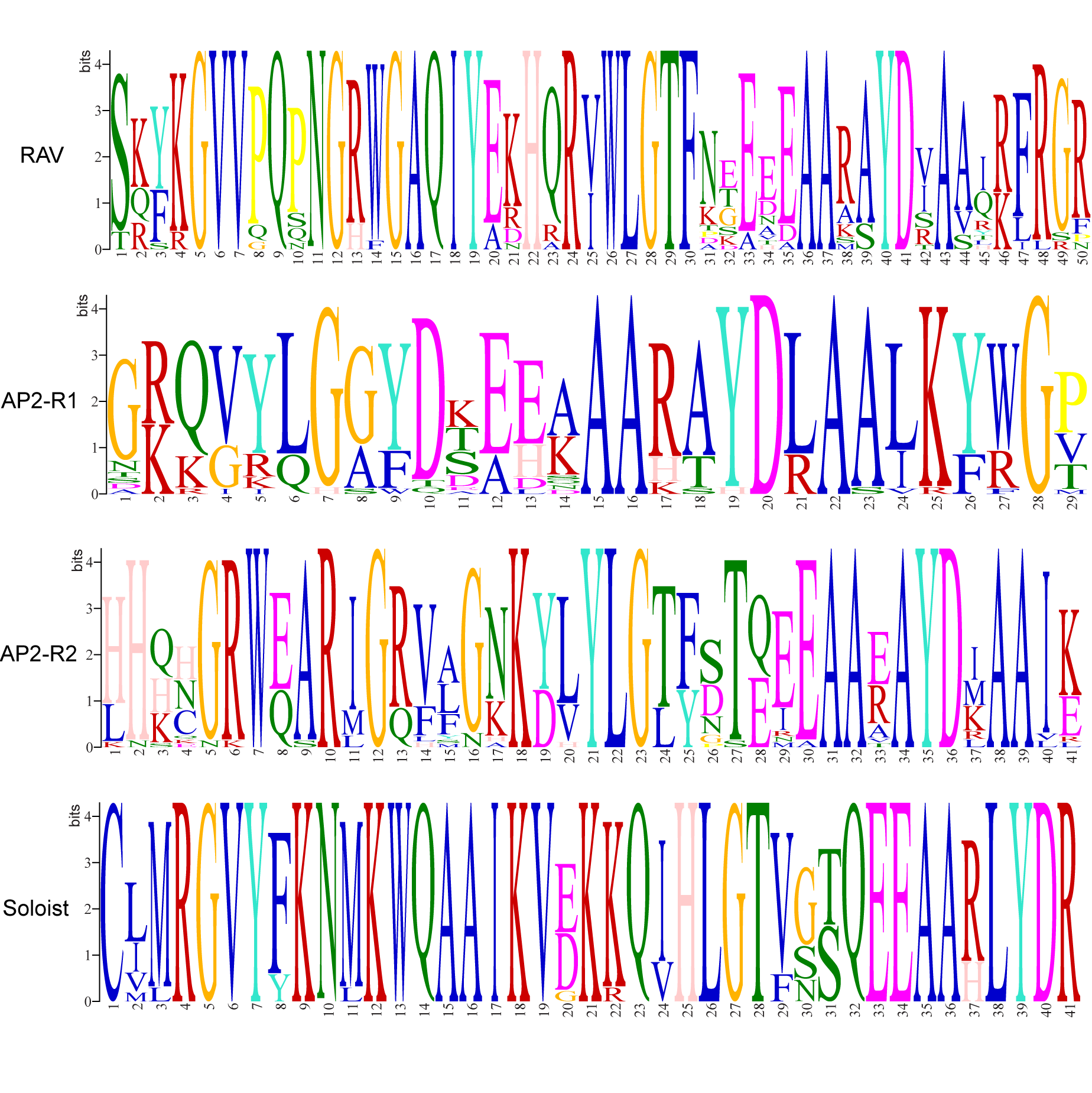

Supplement: Additional file 2: Figure S1 — Phylogenetic tree constructed from the neighbor-joining method using AP2 family transcription factor domains in Chinese cabbage and Arabidopsis. The numbers are bootstrap values based on 1000 iterations. Only bootstrap values larger than 50 are indicated. Figure S2. Phylogenetic tree constructed from the neighbor-joining method using AP2 family transcription factor domains in all 16 species analyzed. Figure S3. AP2/ERF protein motifs from each of the species examined. Figure S4. The ERF subfamily protein motifs derived from each species examined. Figure S5. The DREB subfamily protein motifs derived from each species. Figure S6. The RAV, AP2 and Soloist family protein motifs derived from each species examined. Figure S7. The AP2/ERF superfamily protein motifs derived from each species examined. Figure S8. Comparative analysis of synteny and expansion of AP2/ERF genes. Ten Chinese cabbage and five Arabidopsis chromosome maps were based on the orthologue pair positions, and demonstrate highly conserved synteny. Figure S9. Comparative analysis of synteny and expansion of AP2/ERF genes. Ten Chinese cabbage chromosome maps were based on the paralogue pair positions; and demonstrate highly conserved synteny. Figure S10. The secondary metabolic biosynthesis pathways of the AP2/ERF proteins. Figure S11. The regulatory pathways of the AP2/ERF proteins. Figure S12. The metabolic pathways of the AP2/ERF proteins. Figure S13. AP2/ERF transcription factors classification in Chinese cabbage. The size of each section is proportional to the relative abundance of the AP2/ERF genes assigned to the specific family. Figure S14. Distribution of AP2/ERF transcription factors in various Chinese cabbage tissues. Figure S15. Expression profile cluster analyses from Chinese cabbage DREB subfamily genes. Figure S16. Expression profile cluster analyses from Chinese cabbage RAV family genes. Figure S17. Expression profile cluster analyses from Chinese cabbage AP2 family genes. Figure S18. Chines [file 1471-2164-14-573-S2.zip › Figure S6.png]

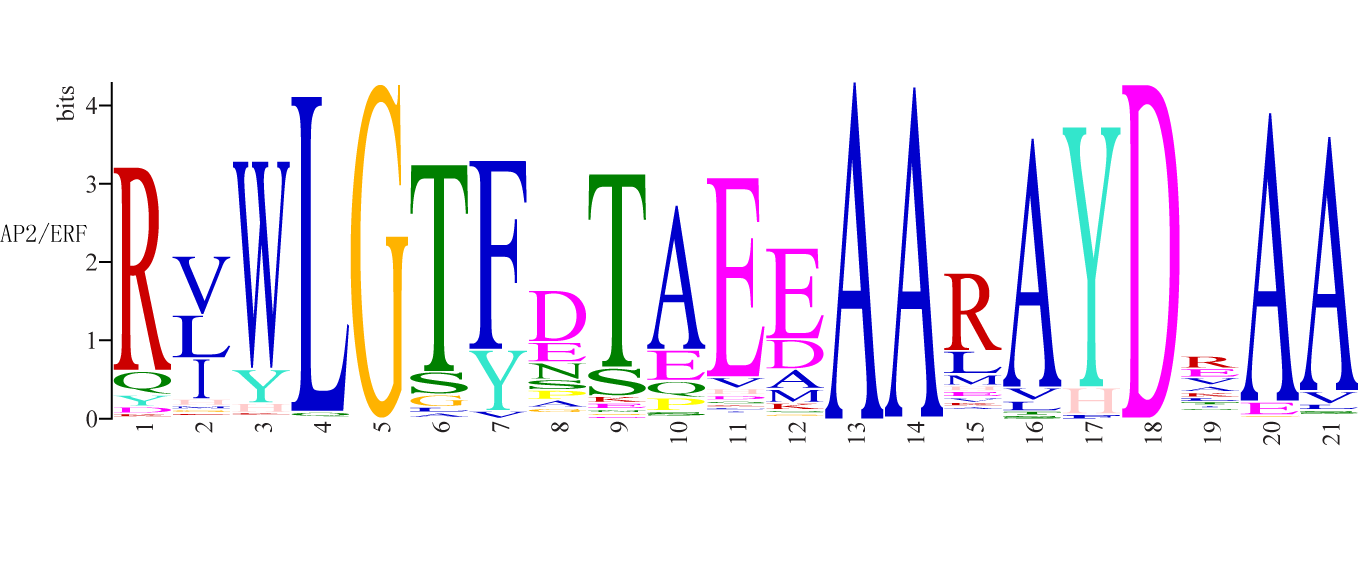

Supplement: Additional file 2: Figure S1 — Phylogenetic tree constructed from the neighbor-joining method using AP2 family transcription factor domains in Chinese cabbage and Arabidopsis. The numbers are bootstrap values based on 1000 iterations. Only bootstrap values larger than 50 are indicated. Figure S2. Phylogenetic tree constructed from the neighbor-joining method using AP2 family transcription factor domains in all 16 species analyzed. Figure S3. AP2/ERF protein motifs from each of the species examined. Figure S4. The ERF subfamily protein motifs derived from each species examined. Figure S5. The DREB subfamily protein motifs derived from each species. Figure S6. The RAV, AP2 and Soloist family protein motifs derived from each species examined. Figure S7. The AP2/ERF superfamily protein motifs derived from each species examined. Figure S8. Comparative analysis of synteny and expansion of AP2/ERF genes. Ten Chinese cabbage and five Arabidopsis chromosome maps were based on the orthologue pair positions, and demonstrate highly conserved synteny. Figure S9. Comparative analysis of synteny and expansion of AP2/ERF genes. Ten Chinese cabbage chromosome maps were based on the paralogue pair positions; and demonstrate highly conserved synteny. Figure S10. The secondary metabolic biosynthesis pathways of the AP2/ERF proteins. Figure S11. The regulatory pathways of the AP2/ERF proteins. Figure S12. The metabolic pathways of the AP2/ERF proteins. Figure S13. AP2/ERF transcription factors classification in Chinese cabbage. The size of each section is proportional to the relative abundance of the AP2/ERF genes assigned to the specific family. Figure S14. Distribution of AP2/ERF transcription factors in various Chinese cabbage tissues. Figure S15. Expression profile cluster analyses from Chinese cabbage DREB subfamily genes. Figure S16. Expression profile cluster analyses from Chinese cabbage RAV family genes. Figure S17. Expression profile cluster analyses from Chinese cabbage AP2 family genes. Figure S18. Chines [file 1471-2164-14-573-S2.zip › Figure S7.png]

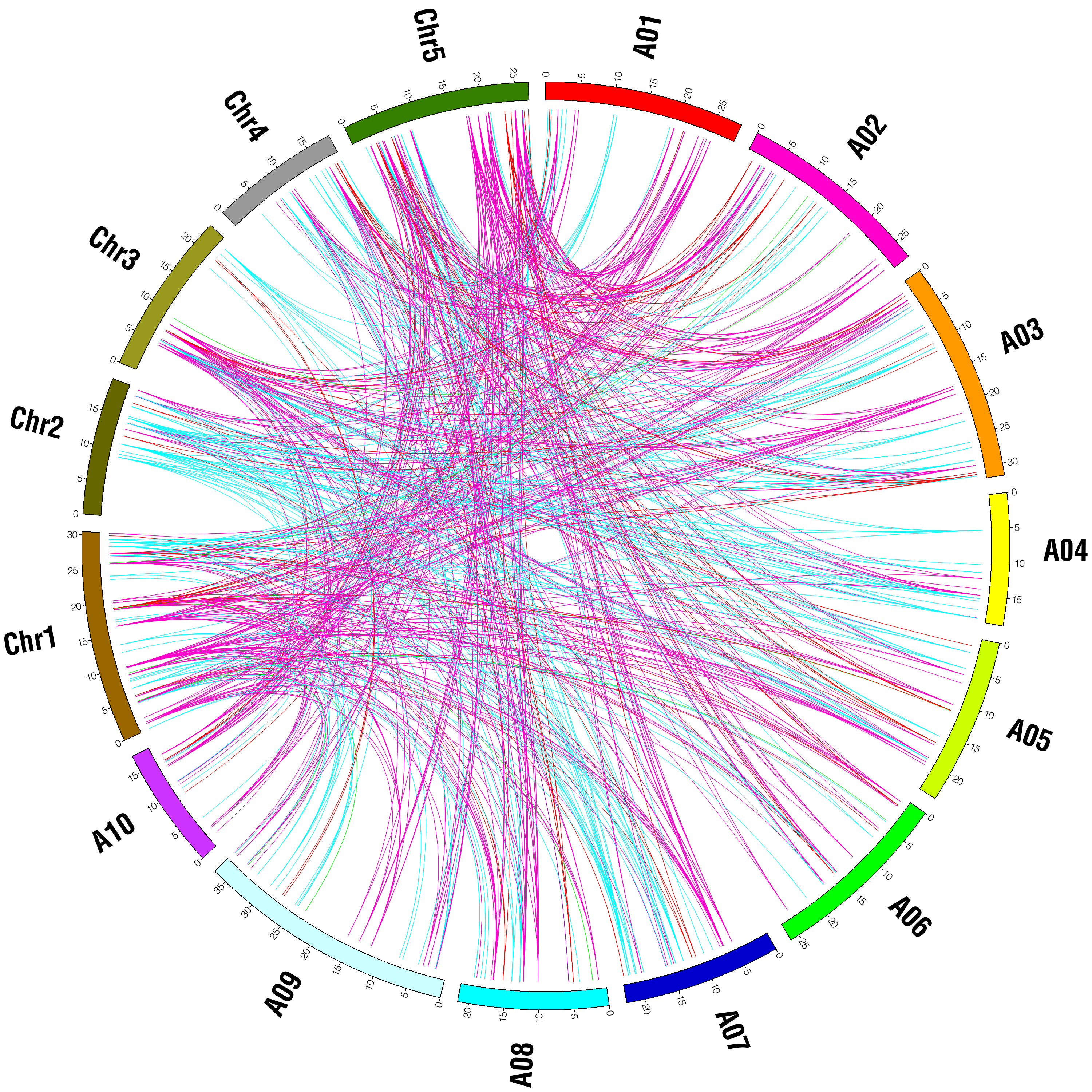

Supplement: Additional file 2: Figure S1 — Phylogenetic tree constructed from the neighbor-joining method using AP2 family transcription factor domains in Chinese cabbage and Arabidopsis. The numbers are bootstrap values based on 1000 iterations. Only bootstrap values larger than 50 are indicated. Figure S2. Phylogenetic tree constructed from the neighbor-joining method using AP2 family transcription factor domains in all 16 species analyzed. Figure S3. AP2/ERF protein motifs from each of the species examined. Figure S4. The ERF subfamily protein motifs derived from each species examined. Figure S5. The DREB subfamily protein motifs derived from each species. Figure S6. The RAV, AP2 and Soloist family protein motifs derived from each species examined. Figure S7. The AP2/ERF superfamily protein motifs derived from each species examined. Figure S8. Comparative analysis of synteny and expansion of AP2/ERF genes. Ten Chinese cabbage and five Arabidopsis chromosome maps were based on the orthologue pair positions, and demonstrate highly conserved synteny. Figure S9. Comparative analysis of synteny and expansion of AP2/ERF genes. Ten Chinese cabbage chromosome maps were based on the paralogue pair positions; and demonstrate highly conserved synteny. Figure S10. The secondary metabolic biosynthesis pathways of the AP2/ERF proteins. Figure S11. The regulatory pathways of the AP2/ERF proteins. Figure S12. The metabolic pathways of the AP2/ERF proteins. Figure S13. AP2/ERF transcription factors classification in Chinese cabbage. The size of each section is proportional to the relative abundance of the AP2/ERF genes assigned to the specific family. Figure S14. Distribution of AP2/ERF transcription factors in various Chinese cabbage tissues. Figure S15. Expression profile cluster analyses from Chinese cabbage DREB subfamily genes. Figure S16. Expression profile cluster analyses from Chinese cabbage RAV family genes. Figure S17. Expression profile cluster analyses from Chinese cabbage AP2 family genes. Figure S18. Chines [file 1471-2164-14-573-S2.zip › Figure S8.png]

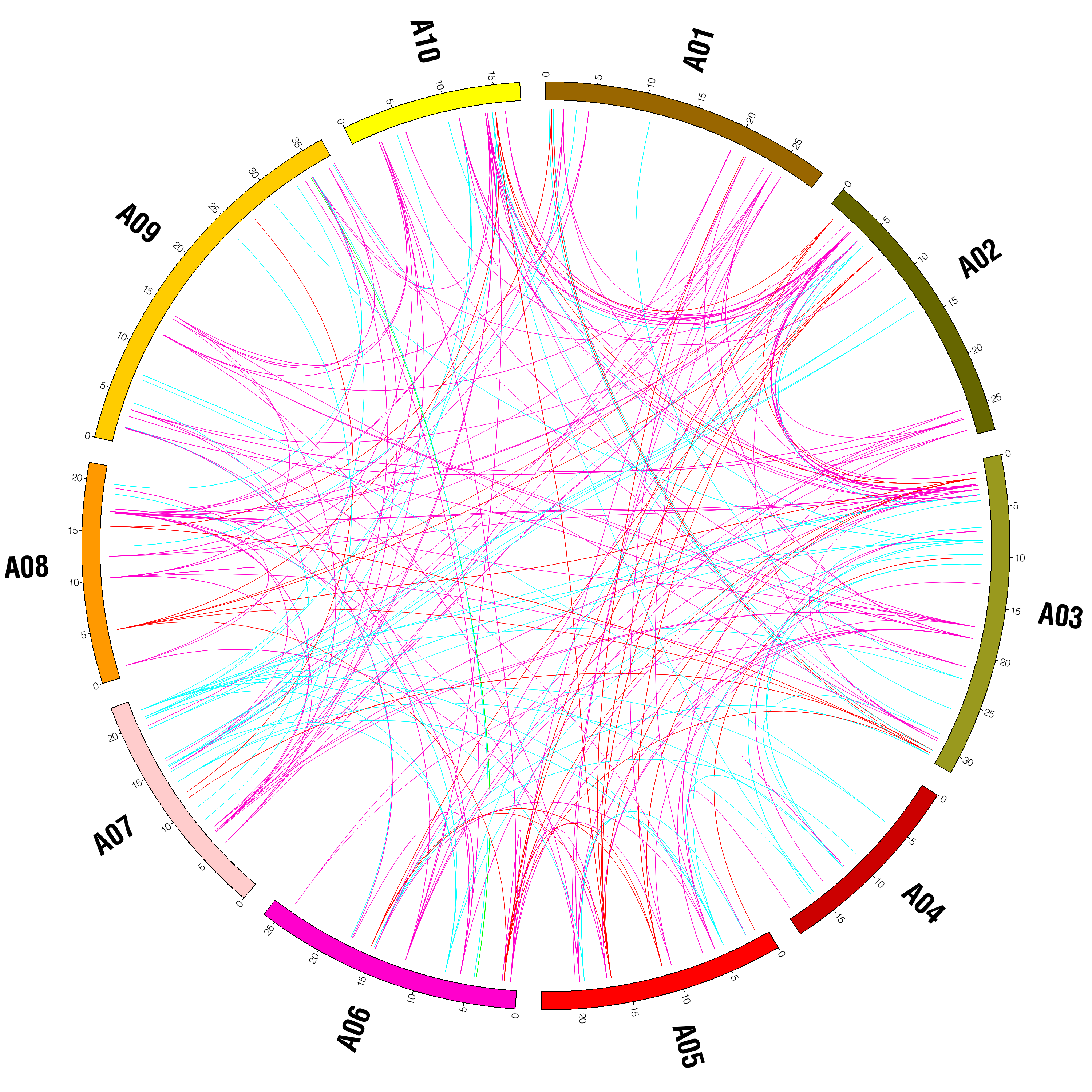

Supplement: Additional file 2: Figure S1 — Phylogenetic tree constructed from the neighbor-joining method using AP2 family transcription factor domains in Chinese cabbage and Arabidopsis. The numbers are bootstrap values based on 1000 iterations. Only bootstrap values larger than 50 are indicated. Figure S2. Phylogenetic tree constructed from the neighbor-joining method using AP2 family transcription factor domains in all 16 species analyzed. Figure S3. AP2/ERF protein motifs from each of the species examined. Figure S4. The ERF subfamily protein motifs derived from each species examined. Figure S5. The DREB subfamily protein motifs derived from each species. Figure S6. The RAV, AP2 and Soloist family protein motifs derived from each species examined. Figure S7. The AP2/ERF superfamily protein motifs derived from each species examined. Figure S8. Comparative analysis of synteny and expansion of AP2/ERF genes. Ten Chinese cabbage and five Arabidopsis chromosome maps were based on the orthologue pair positions, and demonstrate highly conserved synteny. Figure S9. Comparative analysis of synteny and expansion of AP2/ERF genes. Ten Chinese cabbage chromosome maps were based on the paralogue pair positions; and demonstrate highly conserved synteny. Figure S10. The secondary metabolic biosynthesis pathways of the AP2/ERF proteins. Figure S11. The regulatory pathways of the AP2/ERF proteins. Figure S12. The metabolic pathways of the AP2/ERF proteins. Figure S13. AP2/ERF transcription factors classification in Chinese cabbage. The size of each section is proportional to the relative abundance of the AP2/ERF genes assigned to the specific family. Figure S14. Distribution of AP2/ERF transcription factors in various Chinese cabbage tissues. Figure S15. Expression profile cluster analyses from Chinese cabbage DREB subfamily genes. Figure S16. Expression profile cluster analyses from Chinese cabbage RAV family genes. Figure S17. Expression profile cluster analyses from Chinese cabbage AP2 family genes. Figure S18. Chines [file 1471-2164-14-573-S2.zip › Figure S9.png]
